# *N*-acetylglucosamine-6-O-sulfation on intestinal mucins prevents obesity and intestinal inflammation by regulating gut microbiota

**DOI:** 10.1172/jci.insight.165944

**Published:** 2023-08-22

**Authors:** Hirohito Abo, Aoi Muraki, Akihito Harusato, Tetsuya Imura, Maki Suzuki, Kohta Takahashi, Timothy L. Denning, Hiroto Kawashima

**Affiliations:** 1 Laboratory of Microbiology and Immunology, Graduate School of Pharmaceutical Science, Chiba University, Chiba, Japan.; 2 Molecular Gastroenterology and Hepatology, and; 3 Department of Surgical Pathology, Graduate School of Medical Science, Kyoto Prefectural University of Medicine, Kyoto, Japan.; 4 Institute for Biomedical Sciences, Georgia State University, Atlanta, Georgia, USA.

**Keywords:** Gastroenterology, Inflammation, Glycobiology, Mouse models, Obesity

## Abstract

Intestinal mucins play an essential role in the defense against bacterial invasion and the maintenance of gut microbiota, which is instrumental in the regulation of host immune systems; hence, its dysregulation is a hallmark of metabolic disease and intestinal inflammation. However, the mechanism by which intestinal mucins control the gut microbiota as well as disease phenotypes remains nebulous. Herein, we report that *N*-acetylglucosamine (GlcNAc)-6-O-sulfation of O-glycans on intestinal mucins performs a protective role against obesity and intestinal inflammation. *Chst4^–/–^* mice, lacking GlcNAc-6-O-sulfation of the mucin O-glycans, showed significant weight gain and increased susceptibility to dextran sodium sulfate–induced colitis as well as colitis-associated cancer accompanied by significantly reduced immunoglobulin A (IgA) production caused by an impaired T follicular helper cell–mediated IgA response. Interestingly, the protective effects of GlcNAc-6-O-sulfation against obesity and intestinal inflammation depend on the gut microbiota, evidenced by the modulation of the gut microbiota by cohousing or microbiota transplantation reversing disease phenotypes and IgA production. Collectively, our findings provide insight into the significance of host glycosylation, more specifically GlcNAc-6-O-sulfation on intestinal mucins, in protecting against obesity and intestinal inflammation via regulation of the gut microbiota.

## Graphical abstract

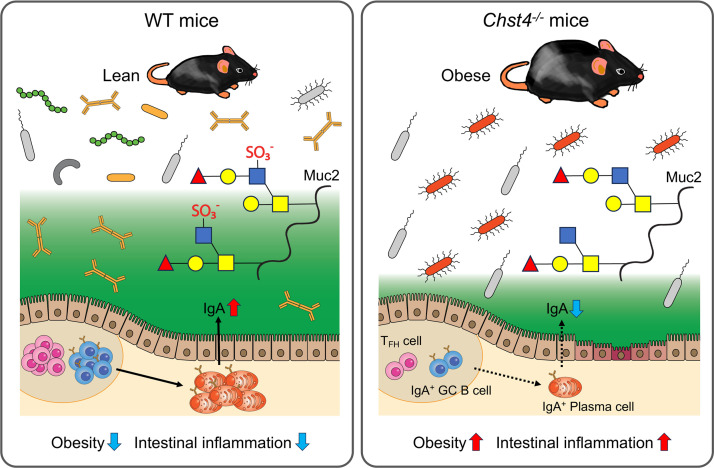

## Introduction

The intestinal microbiota comprises hundreds of trillions of microbes, including bacteria, viruses, fungi, protists, and archaeans, that codevelop with the host throughout its entire life. Gut microbes have significant impact on various aspects of health and disease, including metabolic syndrome and inflammatory bowel disease (IBD) (1, 2).

The emerging relationship between gut microbiota and the host immune system has been explored in the context of multiple inflammatory conditions. Recently, it has become evident that host immune systems positively regulate the gut microbiota. Particularly, immunoglobulin A (IgA) has been reported to be a mucosal immune mediator regulating metabolic disease and intestinal inflammation via its effects on the gut microbiota (3). Conversely, signals conveyed by the microbiome also play a critical role in regulating the host immune system (4). Understanding this bidirectional regulation could provide important insights into the role of the host immune system and gut microbiota in health and diseases.

Intestinal mucins, the most abundant of which is mucin 2 (MUC2), are secreted by goblet cells to form mucus layers in the small and large intestine (5). *Muc2*-deficient mice spontaneously develop colitis and adenoma within the small and large intestine (6, 7). Additionally, MUC2 is highly glycosylated, with O-linked glycans attached to serine or threonine residues having protective roles against intestinal inflammation and tumorigenesis (8–10). These findings highlight the regulatory role of MUC2 and its O-glycans in intestinal immune tolerance and barrier function. Furthermore, mice lacking core 1– and core 3–derived O-glycans exhibit significantly different bacterial compositions compared with wild-type (WT) mice (11). This finding suggests that the O-glycans on mucins modulate the mucosal ecosystem, which comprises various commensals. However, due to the complexity of glycan structures, the specific functional units responsible for inflammatory diseases and microbiota regulation remain unclear.

A distinct structural characteristic of mucin O-glycans is the sulfation of its 6-hydroxyl group of *N*-acetyl-D-glucosamine (GlcNAc-6-O-sulfation), which is the dominant sulfate modification of intestinal mucins (12). We previously identified *N*-acetylglucosamine 6-O-sulfotransferase-2 (GlcNAc6ST-2), which is encoded by *Chst4*, as a primary sulfotransferase that catalyzes GlcNAc-6-O-sulfation (12). However, the function of GlcNAc-6-O-sulfation of mucin O-glycans in vivo remains to be elucidated.

In this study, we define the critical role of the sulfated mucin O-glycans in obesity and intestinal inflammation. *Chst4^–/–^* mice, which lack GlcNAc-6-O-sulfation of mucin O-glycans, developed obesity and were susceptible to experimental colitis as well as colitis-associated cancer (CAC). Moreover, we identified a dramatic change in microbiota composition between WT and *Chst4^–/–^* mice. Furthermore, *Chst4^–/–^* mice exhibited significantly reduced fecal IgA levels, with fewer IgA^+^ germinal center (GC) B cells in Peyer’s patches (PPs) and a lower frequency of T follicular helper (Tfh) cells relative to WT mice. Through cohousing experiments, we demonstrated that the weight gain and increased colitis susceptibility observed in *Chst4^–/–^* mice were highly dependent on microbiota alterations. Interestingly, impaired Tfh cell differentiation completely recovered after cohousing, accompanied by the restoration of IgA-expressing GC B cells. Collectively, our findings define the GlcNAc-6-O-sulfation of mucin O-glycans as a critical regulatory factor for maintaining gut microbiota and host immune systems that prevent obesity and intestinal inflammation.

## Results

### Deficiency of GlcNAc-6-O-sulfation promotes age-associated and high-fat diet–induced obesity.

**Figure 1:**
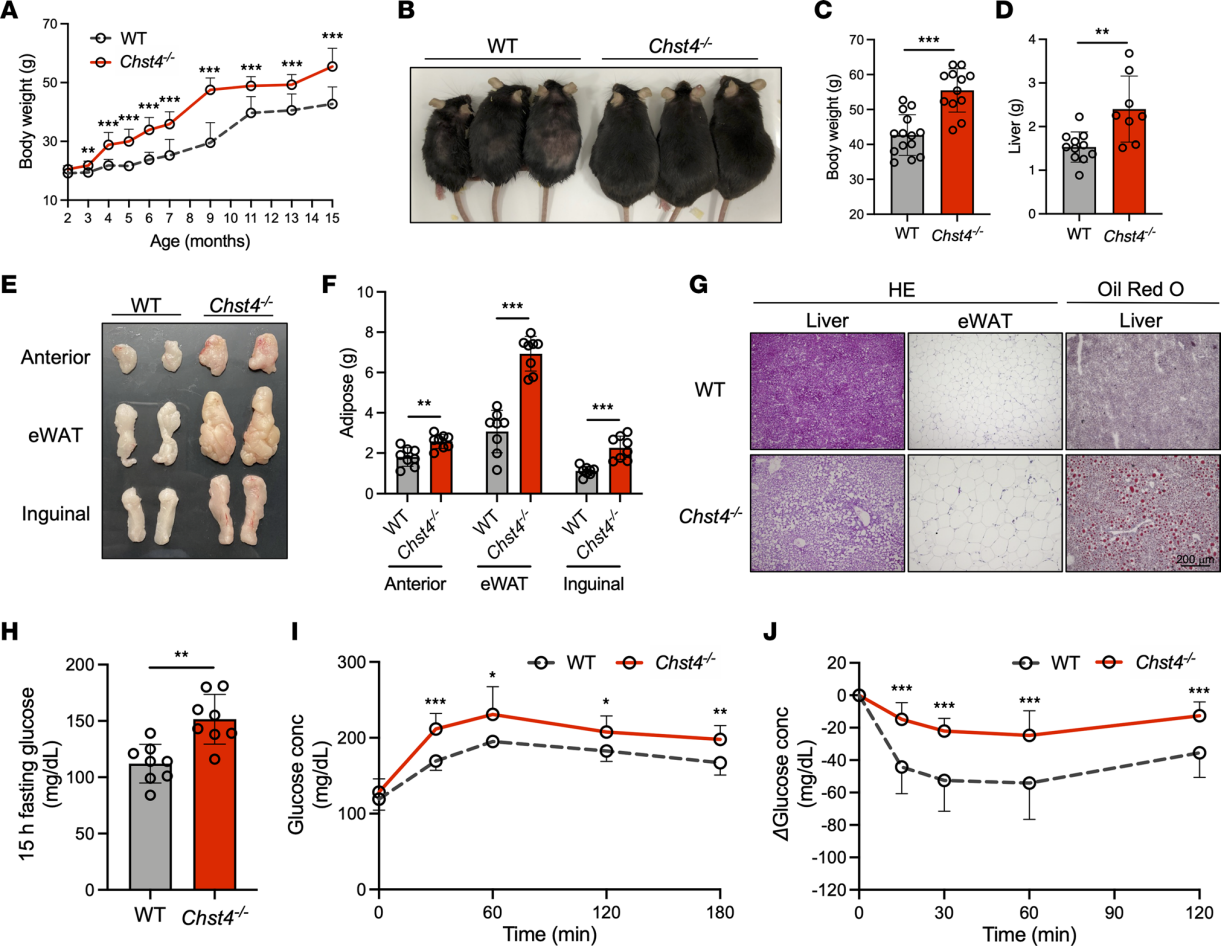
Deficiency of GlcNAc-6-O-sulfation promotes age-associated obesity. (**A**) Weight gain of WT and *Chst4^–/–^* mice measured over a 15-month period. (**B**) Representative image of 15-month-old WT and *Chst4^–/–^* mice. (**C**) Total weight of 15-month-old WT and *Chst4^–/–^* mice (WT *n* = 14, *Chst4^–/–^* *n* = 12). (**D**) Liver weight of WT and *Chst4^–/–^* mice (WT *n* = 11, *Chst4^–/–^* *n* = 8). (**E**) Representative image of epididymal white adipose tissue (eWAT) from WT and *Chst4^–/–^* mice. (**F**) Adipose tissue weight (*n* = 8). (**G**) Representative H&E and Oil Red O staining of liver and adipose tissue from WT and *Chst4^–/–^* mice. Scale bar: 200 μm. (**H**) Fifteen-hour fasting blood glucose concentrations in WT and *Chst4^–/–^* mice at 15 months. (**I**) Blood glucose concentration in glucose tolerance tests (*n* = 8). (**J**) Change in blood glucose concentration in WT and *Chst4^–/–^* mice after additional insulin challenge (WT *n* = 10, *Chst4^–/–^* *n* = 9). Data are representative of 2 independent experiments, and presented as the mean ± SD. *P < 0.05, **P < 0.01, ***P < 0.001 via 2-way ANOVA followed by Tukey’s multiple-comparison test (**A**, **I**, and **J**) or unpaired, 2-tailed *t* test (**C**, **D**, **F**, and **H**).

We previously reported that the protein product of *Chst4*, GlcNAc6ST-2, is the primary sulfotransferase catalyzing the GlcNAc-6-O-sulfation of mucin O-glycans (Supplemental Figure 1, A and B, and Supplemental Figure 2, A and B; supplemental material available online with this article; https://doi.org/10.1172/jci.insight.165944DS1) (12). During housing, we unexpectedly found that 15-month-old *Chst4^–/–^* mice exhibited increased weight gain compared with WT mice (Figure 1, A–C). Despite being fed a normal chow diet, *Chst4^–/–^* mice had a significantly higher liver weight (Figure 1D). Consistent with these findings, we also observed increased fat accumulation in *Chst4^–/–^* mice (Figure 1, E and F). Hematoxylin and eosin (H&E) and Oil Red O staining revealed fatty liver, aberrant adipocyte size, and increased lipid accumulation in *Chst4^–/–^* mice (Figure 1G). Obesity in mice fed a standard mouse chow requires several months to develop. Conversely, when mice were fed high-fat diet (HFD), both male and female mice *Chst4^–/–^* mice exhibited significant increases in weight gain (Supplemental Figure 3, A–D). Furthermore, we observed increased liver weight (Supplemental Figure 3E) and adipose tissue mass (Supplemental Figure 3, F and G) compared with WT mice, as early as 6 weeks after HFD initiation and without any changes in food intake (Supplemental Figure 3H). Moreover, similarly to aged *Chst4^–/–^* mice, HFD-fed *Chst4^–/–^* mice exhibited fatty liver, aberrant adipocyte size, and increased lipid accumulation (Supplemental Figure 3I).

Individuals with obesity develop various metabolic disease comorbidities, including glucose tolerance and insulin resistance. We next examined the blood glucose concentration of WT and *Chst4^–/–^* mice. After a 15-hour fasting, 15-month-old *Chst4^–/–^* mice exhibited significantly increased blood glucose levels relative to age-matched WT mice (Figure 1H). Consistent with this finding, *Chst4^–/–^* mice had an impaired ability to restore blood glucose down to basal levels following glucose challenge (Figure 1I). Moreover, when administered insulin, *Chst4^–/–^* mice exhibited reduced sensitivity relative to WT mice (Figure 1J). After 6 weeks of HFD, we observed similar phenotypes, including elevated fasting blood glucose (Supplemental Figure 3J), higher glucose tolerance (Supplemental Figure 3K), and insulin resistance (Supplemental Figure 3L). Altogether, these findings suggest that deficiency of GlcNAc-6-O-sulfation on mucin O-glycans promotes age-associated as well as diet-induced obesity and metabolic syndrome.

### Chst4^–/–^ mice develop low-grade intestinal inflammation and increased susceptibility to DDS-induced colitis.

**Figure 2:**
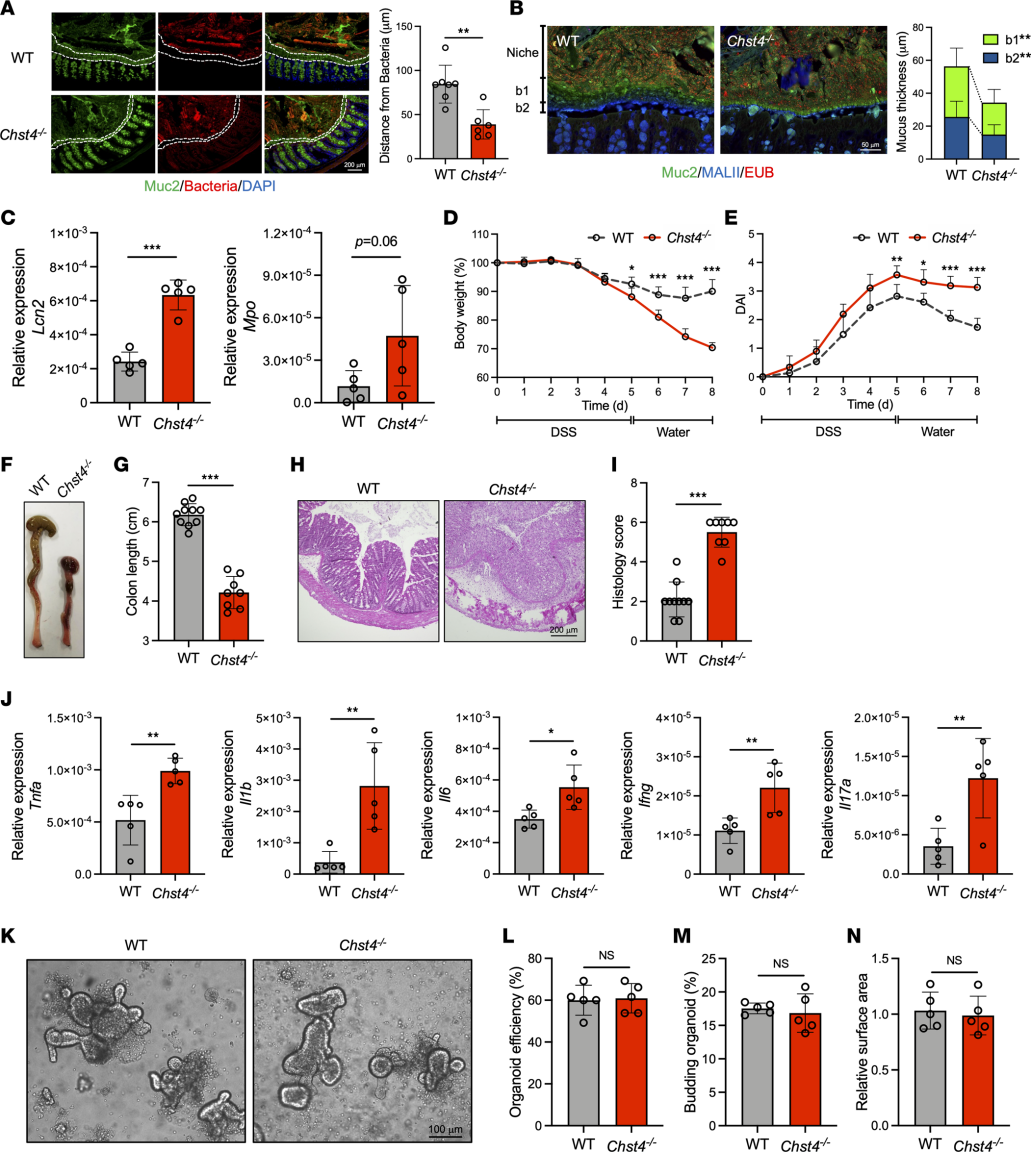
*Chst4^–/–^* mice exhibit low-grade inflammation and increased susceptibility to DSS-induced colitis. (**A**) Analysis of mucus thickness in the colon of 7-week-old healthy WT and *Chst4^–/–^* mice by Carnoy’s fixation (WT *n* = 7, *Chst4^–/–^* *n* = 6). (**B**) Immunofluorescent staining of Carnoy’s-fixed colonic tissues for MUC2, MALII, and EUB (*n* = 15). (**C**) Expression of *Lcn2* and *Mpo* in the colonic tissue of 7-week-old mice (*n* = 5). (**D** and **E**) Weight change and DAI score of mice administered DSS for 5 days, followed by drinking water for 4 days. (**F**) Picture of colons from WT and *Chst4^–/–^* mice. (**G**) Colon lengths of mice shown in **F**. (**H**) Representative H&E-stained colon section and (**I**) histology scores (WT *n* = 10, *Chst4^–/–^* *n* = 8). (**J**) Expression of inflammatory cytokines (*Tnfa*, *Il1b*, *Il6*, *Ifng*, *Il17a*) analyzed using qPCR (*n* = 5). (**K**) Representative pictures of colonoids derived from WT and *Chst4^–/–^* mice. (**L**–**N**) Organoid efficiency, frequency of budding organoids, and surface area of WT and *Chst4^–/–^* mice (*n* = 5). Scale bars: 200 μm (**A** and **H**), 50 μm (**B**), and 100 μm (**K**). Data are representative of 2 independent experiments, and presented as the mean ± SD. NS, not significant, *P < 0.05, **P < 0.01, ***P < 0.001 via unpaired, 2-tailed *t* test (**A**–**C**, **I**, **J**, and **L**–**N**) or 2-way ANOVA followed by Tukey’s multiple-comparison test (**D** and **E**).

Intestinal inflammation is strongly associated with obesity, and low-grade inflammation is a symptom of metabolic syndrome in humans as well as mice (13). We next examined the extent to which *Chst4* deletion contributes to subtle inflammatory phenotypes. In 7-week-old *Chst4^–/–^* mice, bacteria and host epithelial cells were in greater proximity, detected by mucus-preserving Carnoy’s fixation, indicating increased migration of bacteria into the mucus layer than in WT mice (Figure 2A). A recent study revealed that colonic mucus consists of 2 distinct O-glycosylated layers of MUC2, a major form (b1 layer) produced by the proximal colon and a minor form (b2 layer) derived from the distal colon (11). To determine which mucus layer was primarily lost in *Chst4^–/–^* mice, we conducted staining of the colonic tissues with MALII lectin, which recognizes α2,3-linked sialylated and sulfated glycans (14). We found that both of b1 and b2 layers were significantly reduced in *Chst4^–/–^* mice compared with those in WT mice (Figure 2B). In addition, we detected a significant increase in lipocalin 2 (*Lcn2*) expression in large intestinal tissue from *Chst4^–/–^* mice, which serves as a marker of intestinal inflammation (Figure 2C) (15). Another marker, myeloperoxidase (*Mpo*), also showed an increase but was statistically insignificant (Figure 2C). Consistent with upregulation of inflammation markers, we observed an elevated infiltration of immune cells in the colon of *Chst4^–/–^* mice confirmed by H&E staining of littermate male and female controls (Supplemental Figure 4, A and B). These observations indicate that *Chst4^–/–^* mice spontaneously develop low-grade intestinal inflammation.

Since we observed low-grade intestinal inflammation in *Chst4^–/–^* mice, we hypothesized that *Chst4^–/–^* mice might be more sensitive to chemically induced colitis. To explore this, we employed dextran sodium sulfate–induced (DSS-induced) models of colitis, which exhibit intestinal epithelial damage, followed by epithelial repair after DSS is discontinued and replaced with regular drinking water. As expected, *Chst4^–/–^* mice exhibited greater weight loss (Figure 2D) and failed to recover from epithelial injury, as indicated by the greater disease activity index (DAI) score (Figure 2E), decreased colon length (Figure 2, F and G), and higher histology score (Figure 2, H and I). Consistent with these findings, we detected increased mRNA expression of proinflammatory cytokines, including *Tnfa*, *Il1b*, *Il6*, *Ifng*, and *Il17a* (Figure 2J). Next, we examined whether *Chst4^–/–^* mice experienced intestinal epithelial cell (IEC) dysfunction. To this end, organoids of the large intestine, which is a well-established system for characterizing IECs, were generated from WT and *Chst4^–/–^* mice (16). Three indices for evaluating organoid growth — organoid efficiency, budding, and surface area — were not different between WT and *Chst4^–/–^* mice (Figure 2, K–N). Altogether, these findings indicate that GlcNAc-6-O-sulfation of mucin O-glycans is essential for protection against DSS-induced colonic damage without affecting IEC growth.

### Loss of GlcNAc-6-O-sulfation on mucins exacerbates CAC.

**Figure 3:**
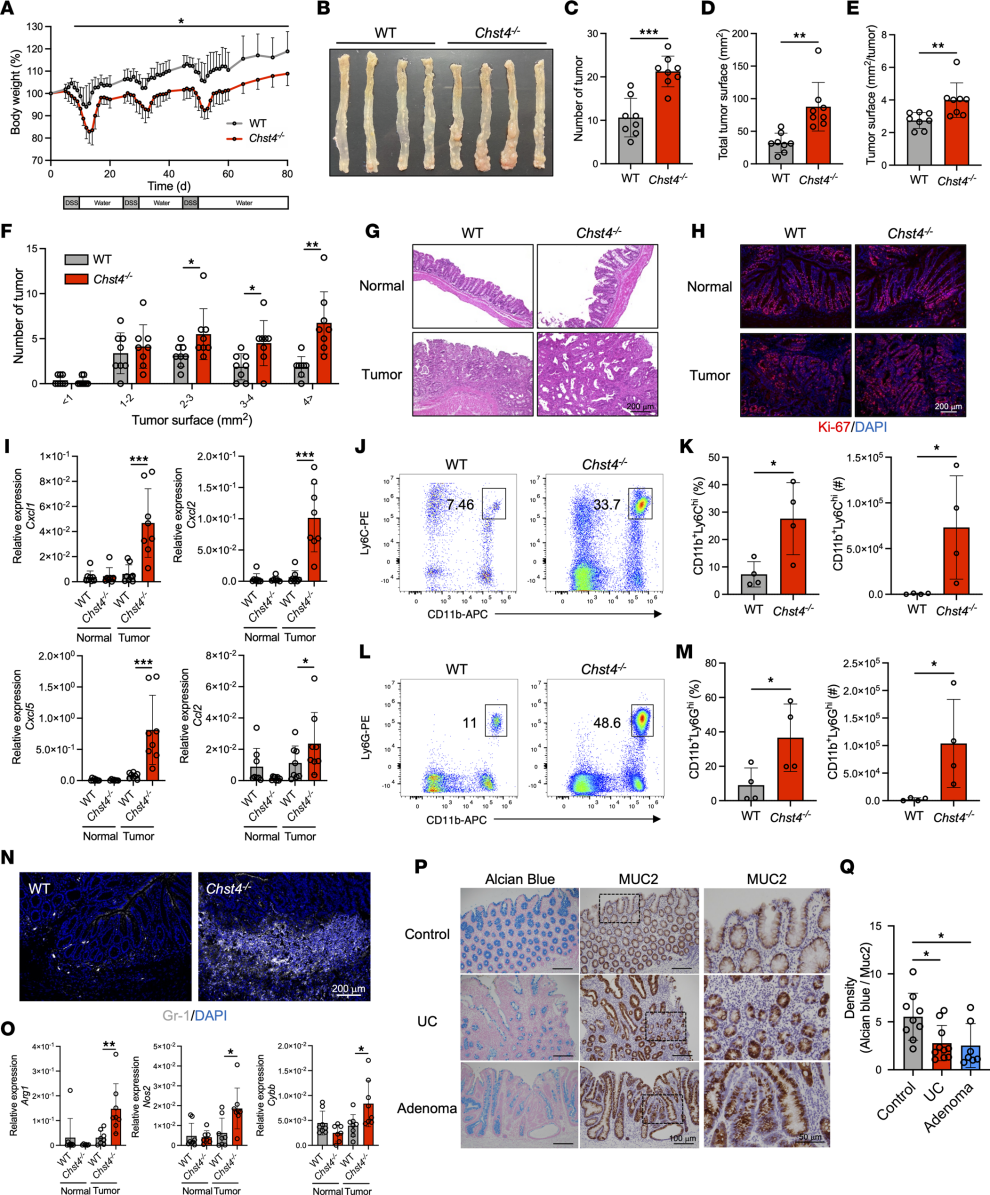
Loss of GlcNAc-6-O-sulfation on mucins exacerbates colitis-associated cancer. (**A**) Weight change of WT and *Chst4^–/–^* mice treated with AOM/DSS. (**B**) Representative pictures of mouse colons. (**C**) Number of tumors in WT and *Chst4^–/–^* mice. (**D**–**F**) Tumor surface area (*n* = 8). (**G**) Representative pictures of H&E-stained colonic tissue from AOM/DSS–treated WT and *Chst4^–/–^* mice. (**H**) Immunofluorescent staining for Ki-67. (**I**) Expression of chemokines related to MDSC infiltration (*n* = 6–8). (**J**) Representative FACS plot of monocyte-derived MDSCs among lamina propria cells isolated from colonic tissue. (**K**) Frequency and cell number shown in **J**. (**L**) Representative FACS plot of granulocyte-derived MDSCs among lamina propria cells isolated from colonic tissue. (**M**) Frequency and cell number shown in **L** (*n* = 4). (**N**) Immunofluorescent staining of colonic tissues with anti–Gr-1 antibody. (**O**) The mRNA expression of *Arg1*, *Nos2*, and *Cybb* in colonic tumor and normal tissue (*n* = 6–8). (**P**) Representative images of human colonic tissue stained with Alcian blue on the left and tissue stained for MUC2 on the right. The square area is shown at higher magnification. (**Q**) Alcian blue intensity normalized to MUC2 staining (healthy *n* = 9, UC *n* = 10, adenoma *n* = 7). Scale bars: 200 μm (**G**, **H**, and **N**), 100 μm (**P**, left and middle columns), and 50 μm (**P**, right column). Data are representative of 2 independent experiments, and presented as the mean ± SD. *P < 0.05, **P < 0.01, ***P < 0.001 via 2-way ANOVA followed by Tukey’s multiple-comparison test (**A**), unpaired, 2-tailed *t* test (**C**–**F**, **K**, and **M**), or 1-way ANOVA (**I**, **O**, and **Q**).

Colonic inflammation is a strong risk factor for the development of CAC (17). We next examined the role of GlcNAc-6-O-sulfation on intestinal mucins in a mouse model of CAC established via azoxymethane (AOM) injection followed by 3 cycles of 1.5% DSS treatment. During each cycle of DSS treatment, *Chst4^–/–^* mice exhibited significant body weight loss compared with WT mice (Figure 3A). At the end of the experiment, *Chst4^–/–^* mice developed a greater number of tumors (Figure 3, B and C). Comparable differences were also noted for total and individual tumor surface area (Figure 3, D–F). Histological analysis revealed lower-grade adenomas in WT mice relative to *Chst4^–/–^* mice (Figure 3G). Moreover, we detected more Ki-67^+^ cells in the tumors of AOM/DSS–treated *Chst4^–/–^* mice relative to those of WT mice (Figure 3H). Taken together, these findings indicate that GlcNAc-6-O-sulfation has a protective role against CAC.

It has been well established that myeloid-derived suppressor cell (MDSC) infiltration into the intestinal tumor microenvironment promotes CAC (18, 19). Considering the enhanced tumorigenesis observed in *Chst4^–/–^* mice, we analyzed monocyte- and granulocyte-derived MDSCs in mouse tumors. Using qPCR, we detected increased mRNA expression of chemokines related to MDSC infiltration, including *Cxcl1*, *Cxcl2*, *Cxcl5*, and *Ccl2* (Figure 3I). Consistent with increased chemokine expression, *Chst4^–/–^* mice exhibited higher infiltration of both monocyte- and granulocyte-derived MDSCs, defined as CD11b^+^Ly6C^+^ and CD11b^+^Ly6G^+^, respectively (Figure 3, J–M). Additionally, we observed an enhanced infiltration of MDSCs in the colon of *Chst4^–/–^* mice evidenced by immunofluorescent staining using an anti–Gr-1 antibody, which detects Ly6G and Ly6C (Figure 3N). MDSCs are reported to suppress antitumor immunity via expression of effector molecules such as arginase-1 (*Arg1*), inducible nitric oxide synthase (*Nos2*), and Nox2 (*Cybb*) (20). We detected significant upregulation of these molecules within the tumors of *Chst4^–/–^* mice (Figure 3O). Altogether, these findings demonstrate that GlcNAc-6-O-sulfation deficiency leads to enhanced infiltration of MDSCs into intestinal tissue and causes increased tumorigenesis.

To validate our findings in clinical samples, we evaluated glycan sulfation on the mucosal surface of patients with ulcerative colitis (UC) and colon cancer using Alcian blue staining. While mucosal sulfation was previously reported to be significantly reduced in patients with UC, it is unclear whether this reduction was due to lower sulfation or decreased MUC2 expression, as the latter has also been noted in patients with UC and colon cancer (21, 22). We stained patient colonic tissue using Alcian blue and anti-MUC2 antibodies, quantifying Alcian blue intensity relative to that of MUC2 staining. We detected less sulfation in samples from patients with UC and colon cancer compared with healthy control samples (Figure 3, P and Q). These findings reveal that mucosal glycan sulfation is reduced relative to MUC2 expression, suggesting that insufficient sulfation of mucin O-glycans may be involved in exacerbation of UC and colon cancer.

### The gut microbiota is altered in Chst4^–/–^ mice.

**Figure 4:**
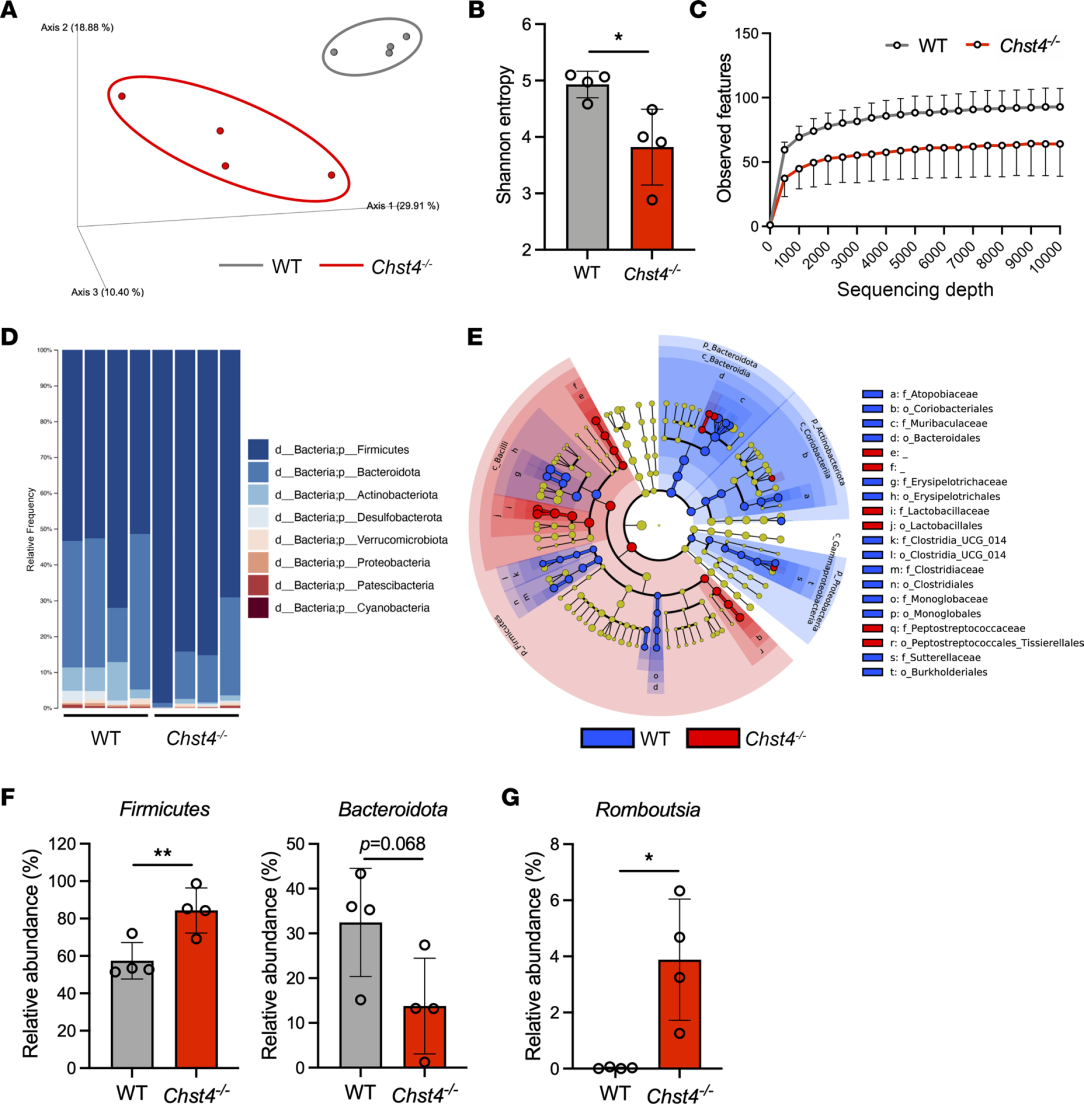
The gut microbiota is altered in *Chst4^–/–^* mice. (**A**) PCoA of ileal microbiota based on unweighted UniFrac distances. (**B** and **C**) Microbiota α diversity as Shannon entropy and observed features. (**D**) Relative abundance of bacteria at the phylum level. (**E**) LEfSe analysis of microbial taxa that were significantly different between WT and *Chst4^–/–^* mice (*n* = 4). (**F**) Relative abundance of *Firmicutes* and *Bacteroidota* in ileal samples from WT and *Chst4^–/–^* mice. (**G**) Relative abundance of *Romboutsia* in ileal samples from WT and *Chst4^–/–^* mice. Data are presented as the mean ± SD. *P < 0.05, **P < 0.01 via unpaired, 2-tailed *t* test (**B**, **F**, and **G**).

Mucin O-glycans were recently described as substrates of mucin-degrading members among the gut microbiota (23, 24). Some members of the human gut microbiome were reported to utilize sulfated glycans as a nutrient source (25). Prompted by these findings and the significant role of intestinal microbiota in the development of obesity and intestinal inflammation, we investigated via 16S V3-V4 rRNA sequencing whether *Chst4* deficiency alters the intestinal microbiota in 7-week-old mice fed with normal chow. Principal coordinates analysis (PCoA) with unweighted UniFrac distance revealed that mucin O-glycan sulfation deficiency led to changes in ileal and fecal microbiota composition between WT (gray dots) and *Chst4^–/–^* (red dots) mice (Figure 4A and Supplemental Figure 5A). Shannon index analysis indicated that the ileal microbiota of *Chst4^–/–^* mice had lower α diversity, which is generally considered a feature of dysbiosis (Figure 4, B and C). These findings indicate that GlcNAc-6-O-sulfation on mucin O-glycans contributes to the maintenance of microbiota composition and diversity.

Changes in microbiota composition, including microbial diversity, are associated with several Western diet–related diseases, such as metabolic syndrome and IBD (26–28). At the phylum level, we observed clear differences in ileal and fecal microbiota between WT and *Chst4^–/–^* mice (Figure 4D and Supplemental Figure 5B). To assess microbiota alteration in greater detail, we performed linear discriminant analysis effect size (LEfSe) analysis, which revealed that multiple amplicon sequence variants (ASVs) in the ileal and fecal microbiota were significantly different between WT and *Chst4^–/–^* mice (Figure 4E and Supplemental Figure 5C). Importantly, we observed an increased abundance of *Firmicutes* and less *Bacteroidota*, a microbiota feature of obesity, at the phylum level in the ileal microbiota of *Chst4^–/–^* mice (Figure 4F) (29). At the family level, *Peptostreptococcaceae*, which is associated with intestinal inflammation, was significantly increased in feces of *Chst4^–/–^* mice (Supplemental Figure 5D) (30). Furthermore, relative abundance of *Prevotellaceae*, which is increased in individuals with obesity and IBD, was increased in feces of *Chst4^–/–^* mice (Supplemental Figure 5E) (31, 32). The genus *Romboutsia*, which is increased in patients with obesity and associated with body weight gain, was also increased in the ileal microbiota of *Chst4^–/–^* mice (Figure 4G) (33). In addition, we found an increased abundance of *Bacteroides* in feces of *Chst4^–/–^* mice, which are well-established mucin-degrading bacteria (Supplemental Figure 5F) (34, 35). Their overabundance may impair mucosal barrier function, leading to increased intestinal inflammation and tumorigenesis.

In order to obtain direct evidence that the deficiency of GlcNAc-6-O-sulfation on mucins was the primary cause of dysbiosis, we conducted a mucus add-back experiment. We isolated mucins from the colons of WT mice, and they were validated by staining with periodic acid–Schiff (PAS) and Alcian blue (pH 1.0) (Supplemental Figure 6A). *Chst4^–/–^* mice were administered mucins via gastric gavage every other day over the course of 2 weeks, after which feces were collected and bacterial composition was analyzed by 16S rRNA sequencing. PCoA revealed a clear difference in the composition of the gut microbiota between the mucin-administered *Chst4^–/–^* mice and *Chst4^–/–^* mice (Supplemental Figure 6B). Upon focusing on individual bacteria, we observed that certain bacteria exhibited an increase (Supplemental Figure 6C) or decrease (Supplemental Figure 6D) in abundance, approaching the levels observed in WT mice, following the administration of mucins derived from WT mice. Altogether, mucin GlcNAc-6-O-sulfation plays a significant role in the maintenance of gut microbiota, suggesting a potential antiinflammatory function.

### GlcNAc-6-O-sulfation deficiency is accompanied by altered antibody class switching and reduced intestinal IgA production caused by impaired Tfh cell/GC B cell axis.

**Figure 5:**
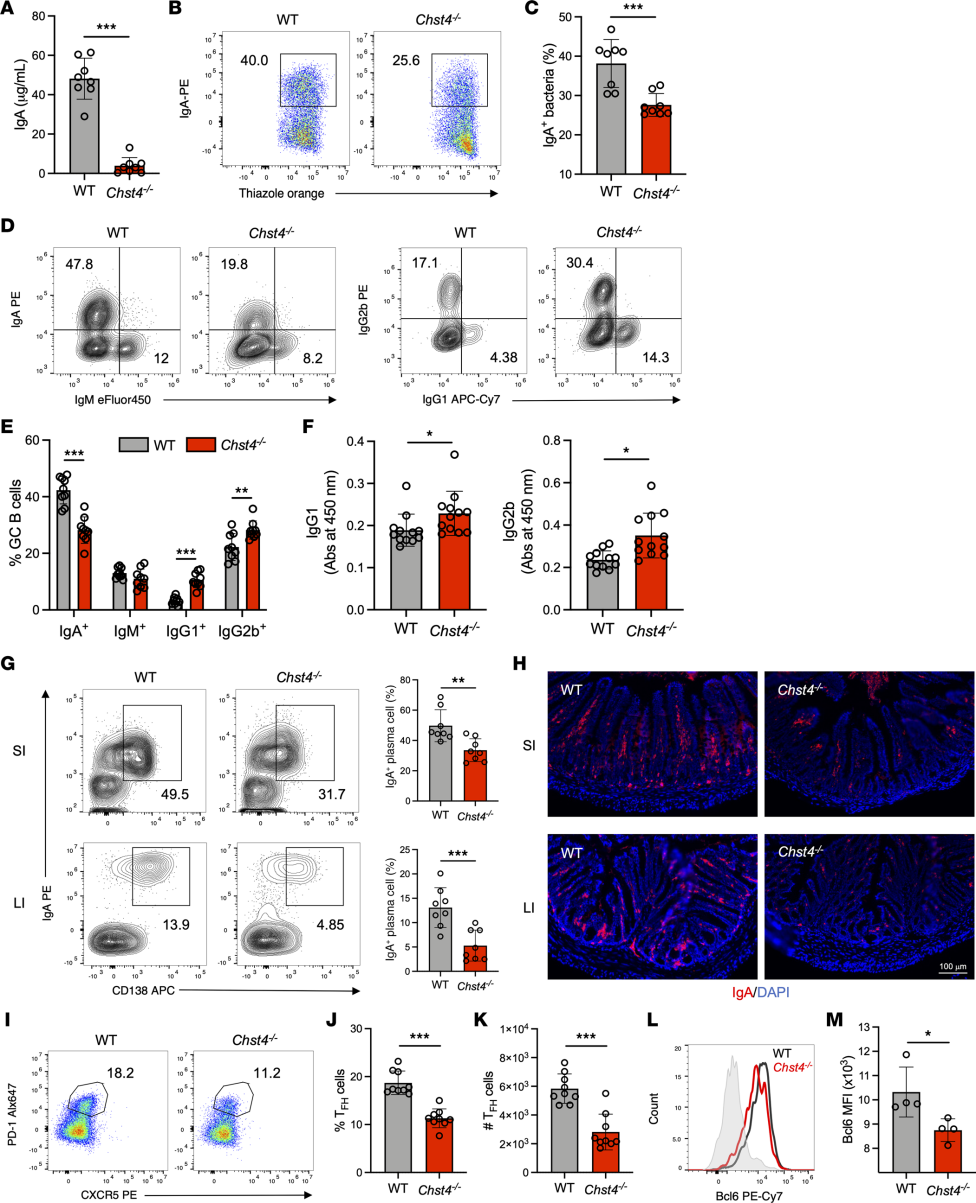
Deficiency of GlcNAc-6-O-sulfation is accompanied by altered antibody class switching and reduced intestinal IgA production caused by an impaired Tfh cell/GC B cell axis. (**A**) Soluble IgA in feces was measured by ELISA. (**B** and **C**) Frequency of IgA-binding bacteria was determined by flow cytometry (*n* = 8). (**D**) Representative flow cytometry plot of GC B cells, which are defined as CD45^+^CD19^+^Fas^+^GL7^+^, from WT and *Chst4^–/–^* mice. (**E**) Frequency of IgA^+^, IgM^+^, IgG1^+^, and IgG2b^+^ GC B cells shown in **D** (*n* = 9). (**F**) Measurement of fecal IgG1 and IgG2b by ELISA. (**G**) Representative flow cytometry plot and frequency of plasma cells in the small and large intestine, defined as CD45^+^B220^–^CD138^+^IgA^+^ (*n* = 8). (**H**) Immunofluorescent staining of IgA in the small (SI) and large intestine (LI). Scale bar: 100 μm. (**I**) Representative flow cytometry plot of Tfh cells defined as CD45^+^CD4^+^CD25^–^CD44^+^PD-1^+^CXCR5^+^. (**J**) Frequency of Tfh cells shown in **H**. (**K**) Absolute cell number of Tfh cells shown in **H** (*n* = 9). (**L**) Expression of Bcl6 in Tfh cells was analyzed using flow cytometry. (**M**) MFI of Bcl6 shown in **K** (*n* = 4). Data are representative of 2 independent experiments, and presented as the mean ± SD. *P < 0.05, **P < 0.01, ***P < 0.001 via unpaired, 2-tailed *t* test.

Previous findings suggest that some members of the human gut microbiota are unable to utilize sulfated mucin oligosaccharides (36). Therefore, the lack of sulfated O-glycans in *Chst4^–/–^* mice could directly affect the gut microbiota composition. Interestingly, we uncovered additional mechanisms that could alter microbial composition. Intestinal IgA is well known to regulate the gut microbial landscape, and IgA-deficient mice harbor microbial dysbiosis that contributes to their increased susceptibility to colitis (37–39). Furthermore, IgA deficiency promotes symptoms of metabolic syndrome (3). Prompted by these findings, we next quantified soluble IgA in feces from WT and *Chst4^–/–^* mice. Compared with WT mice, *Chst4^–/–^* mice had significantly lower amounts of fecal IgA (Figure 5A). Consistent with this finding, we observed a lower frequency of IgA-binding fecal bacteria in *Chst4^–/–^* mice compared with WT mice (Figure 5, B and C). Since PPs are the major lymphoid tissues and the dominant source of IgA-producing cells (40), we hypothesized that mucin sulfation deficiency affects GC reactions to suppress IgA class switching within PPs, especially in GC B cells. As expected, IgA-expressing GC B cells were decreased in *Chst4^–/–^* mice compared with WT mice (Figure 5, D and E), without any differences in the total B cell and GC B cell populations (Supplemental Figure 7, A–D). Conversely, IgG1- and IgG2b-expressing GC B cells were significantly increased in *Chst4^–/–^* mice (Figure 5, D and E). Consistent with this finding, fecal IgG1 and IgG2b were significantly increased in *Chst4^–/–^* mice (Figure 5F). In contrast, analysis of GC B cells in the mesenteric lymph nodes revealed no change in B cell frequency, including that of IgA-expressing GC B cells (Supplemental Figure 7, E–H). We then explored the frequency of IgA-expressing plasma cells, which differentiate from GC B cells and migrate into small and large intestinal tissue. Fewer IgA^+^ plasma cells were observed in the small and large intestinal tissue of *Chst4^–/–^* mice compared with WT mice, as determined using flow cytometry analysis (Figure 5G). Consistent with these findings, decreased IgA staining was observed in small and large intestinal tissue of *Chst4^–/–^* mice compared with those from WT mice (Figure 5H). Altogether, these findings indicate that *Chst4* deficiency affects the antibody class switching of GC B cells, especially in the PPs, resulting in reduced IgA production, which could alter the microbial composition in *Chst4^–/–^* mice.

Next, we explored the specific immune cell subsets implicated in the impaired IgA production observed in *Chst4^–/–^* mice. T cell–dependent and –independent cascades are recognized as drivers of IgA production (40–43). A previous study revealed that competent T cell–dependent immunity, especially Tfh cell–mediated immunity, is required for appropriate IgA production and preventing obesity (44). Furthermore, adoptive transfer of Tfh cells significantly ameliorates the severity of DSS-induced colitis (45). Prompted by these findings, we explored whether *Chst4* deficiency affects Tfh cells, which promote IgA class switching in GCs within PPs. Tfh cells are defined as CD44^+^CD25^–^PD-1^+^CXCR5^+^CD4^+^ T cells. Their frequency and absolute numbers were significantly decreased in *Chst4^–/–^* mice (Figure 5, I–K). Expression of transcription factor Bcl6 in T cells drives Tfh cell specialization (46). We detected decreased protein levels of Bcl6 in *Chst4^–/–^* mice compared with WT mice (Figure 5, L and M). Tfh cells promote IgA expression in the PPs via release of cytokines, including IL-21 and IL-10 (47–49). *Chst4^–/–^* mice exhibited lower expression of these cytokines within the PPs (Supplemental Figure 7I). These data suggest that deficiency of GlcNAc-6-O-sulfation is associated with defects in Tfh cell differentiation, which in turn leads to reduced IgA production in intestinal tissues.

### Altered microbiota accelerates weight gain and intestinal inflammation in Chst4^–/–^ mice.

**Figure 6:**
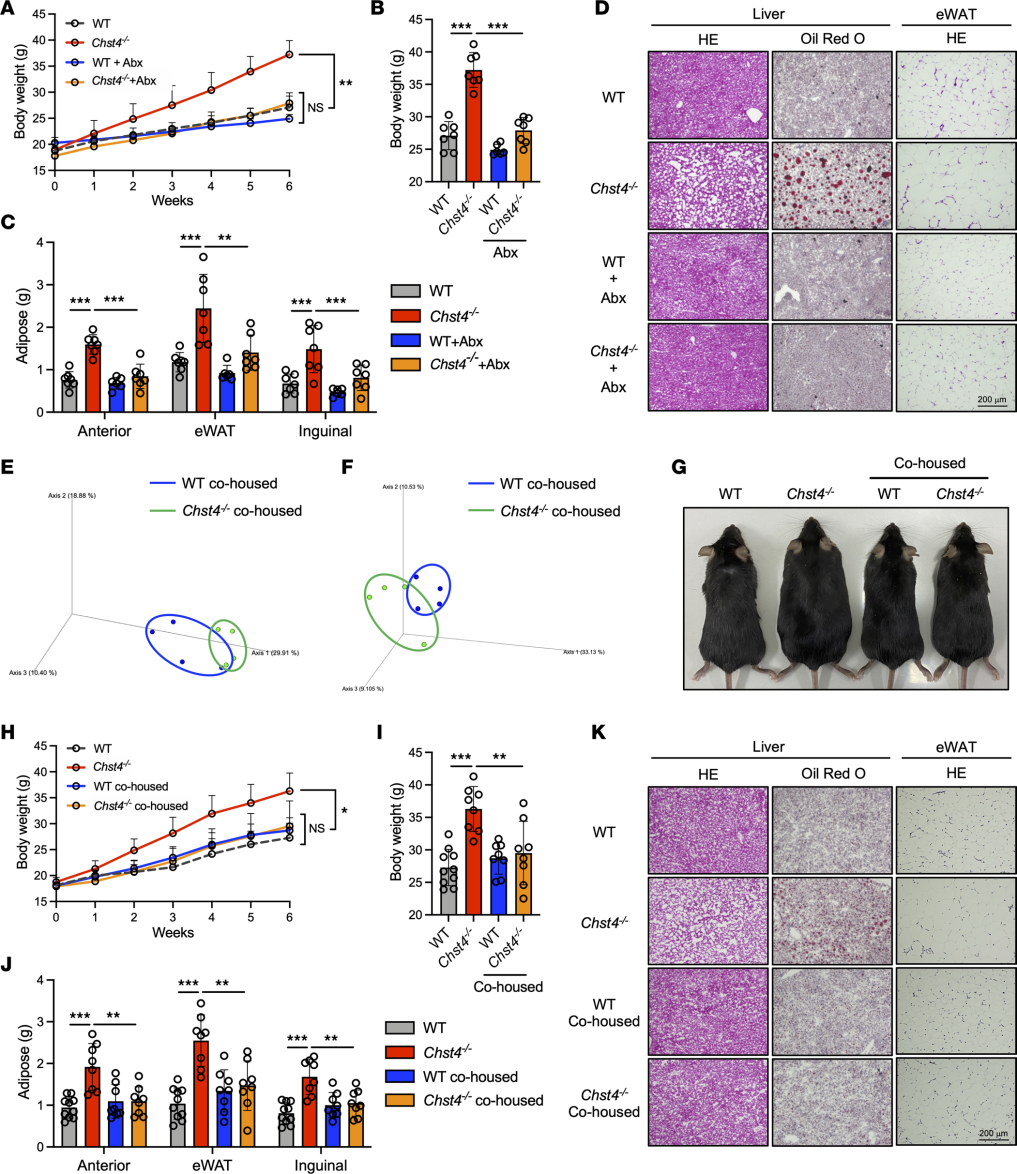
Altered microbiota accelerates weight gain in *Chst4^–/–^* mice. (**A**) Weight gain of WT and *Chst4^–/–^* mice treated with antibiotics (Abx) measured for 6 weeks. (**B**) Total weight of WT and *Chst4^–/–^* mice fed an HFD with antibiotics. (**C**) Adipose tissue weight in WT and *Chst4^–/–^* mice (WT *n* = 7, WT + Abx *n* = 6, *Chst4^–/–^* *n* = 7, *Chst4^–/–^* + Abx *n* = 7). (**D**) Representative H&E and Oil Red O staining of liver and adipose tissue from WT and *Chst4^–/–^* mice treated with antibiotics. (**E**) PCoA analysis of ileal bacteria using unweighted UniFrac distance, after 3 weeks of cohousing. (**F**) PCoA analysis of fecal bacteria using unweighted UniFrac distance, after 3 weeks of cohousing (*n* = 4). (**G**) Representative images of 6-week-old WT and *Chst4^–/–^* mice fed an HFD in the cohousing experiment. (**H**) Weight gain measured over 6 weeks of HFD feeding with cohousing. (**I**) Total weight of WT and *Chst4^–/–^* mice. (**J**) Adipose tissue weight (WT *n* = 10, WT/cohoused *n* = 8, *Chst4^–/–^* *n* = 8, *Chst4^–/–^*/cohoused *n* = 8). (**K**) Representative images of Oil Red O– and H&E-stained liver tissues from WT and *Chst4^–/–^* mice after 6 weeks of HFD feeding with cohousing. Scale bars: 200 μm. Data are representative of 2 independent experiments, and presented as the mean ± SD. *P < 0.05, **P < 0.01, ***P < 0.001 via 2-way ANOVA followed by Tukey’s multiple-comparison test (**A** and **H**) or 1-way ANOVA (**B**, **C**, **I**, and **J**). eWAT, epididymal white adipose tissue.

Microbiota dysregulation is a well-known contributor and feature of metabolic syndrome in humans (50, 51). Considering greater weight gain and symptoms of metabolic syndrome in *Chst4^–/–^* mice, we sought to determine whether microbiota was essential for the observed weight gain. We treated mice with broad-spectrum antibiotics, including ampicillin, neomycin, and vancomycin, while they received an HFD for 6 weeks. WT mice exhibited no difference in weight gain with antibiotic treatment. Conversely, the weight gain observed in *Chst4^–/–^* mice was completely impeded upon antibiotic treatment (Figure 6, A and B). Consistent with these findings, gut microbiota depletion reduced adipose mass of *Chst4^–/–^* mice to the same level of antibiotic-treated WT mice (Figure 6C). The observed hepatic lipid accumulation and aberrant adipocyte size in *Chst4^–/–^* mice were completely rescued by antibiotic treatment (Figure 6D). These findings indicate that the obese phenotype of *Chst4^–/–^* mice is a consequence of their inability to manage their microbiota.

Given that the microbiota maintained by mucin O-glycan sulfation protected against HFD-induced obesity, we next explored whether their modulation by cohousing experiments would affect the observed phenotypes. As mice are coprophagic, cohousing allows for the transfer of bacteria between WT and *Chst4^–/–^* mice. WT and *Chst4^–/–^* mice were housed together with mice of the same or the other genotype from 3 weeks of age. Three weeks of cohousing caused changes in ileal and fecal microbiota, which were distinct from those previously observed in separated WT and *Chst4^–/–^* mice (Figure 6, E and F). After 3 weeks of cohousing, we started HFD feeding and monitored weight gain for 6 weeks. Compared with the separated *Chst4^–/–^* mice, cohoused *Chst4^–/–^* mice exhibited weight gain reduction down to the levels of WT and cohoused WT mice (Figure 6, G–I). Consistently, the greater adipose mass and symptoms of metabolic syndrome, including lipid accumulation, fatty liver, and aberrant adipocyte size, in *Chst4^–/–^* mice were completely prevented by cohousing with WT mice (Figure 6, J and K). Next, we performed LEfSe analysis to compare the composition of gut microbiota between separated and cohoused *Chst4^–/–^* mice. First, in the small intestine, we observed decreased *Firmicutes* and increased *Bacteroidota* in cohoused *Chst4^–/–^* mice, indicating an improvement of the characteristics observed in obese mice (Supplemental Figure 8, A and C). In addition, we found that cohousing led to changes in important gut microbes. *Alistipes*, a genus that is enriched in healthy individuals compared with obese patients, also increased in cohoused mice (Supplemental Figure 8B) (52). *Alistipes* also has an inhibitory role against DSS-induced colitis (53, 54). Moreover, *Parabacteroides goldsteinii*, which has been reported to suppress both obesity and colitis, was increased in cohoused *Chst4^–/–^* mice. Finally, *Bacteroides caecimuris*, frequently identified in patients with nonalcoholic fatty liver disease, was also decreased as a result of cohousing (Supplemental Figure 8B) (55, 56). Hence, the changes in gut microbiota caused by cohousing might have led to an increase in beneficial bacteria, thereby improving the obese phenotypes.

Next, we investigated whether *Chst4^–/–^* mice regain the susceptibility to HFD-induced obesity when they were separated again after cohousing. After 3 weeks of cohousing, mice were separated and housed for an additional 3 weeks (Supplemental Figure 9A). After 6 weeks of being fed an HFD, the body weight of *Chst4^–/–^* mice was found to be markedly reduced during the cohousing period. However, upon reisolation and individual housing, their body weight increased (Supplemental Figure 9, B and C). Consistent with these observations, the weight of adipose tissues, accumulation of lipid in liver, and adipose size were increased upon the reseparation of *Chst4^–/–^* mice (Supplemental Figure 9, D–H). These findings suggest that GlcNAc-6-O-sulfation is essential for the growth of beneficial bacteria and highly contributes to the maintenance of gut microbiota.

Next, to provide further evidence for the potential of microbiota in promoting weight gain in *Chst4^–/–^* mice, we performed microbiota transfer, wherein WT mice were treated with antibiotics and received ileal bacteria from WT and *Chst4^–/–^* mice (Supplemental Figure 10A). Transfer of bacteria from *Chst4^–/–^* mice resulted in significant weight gain and symptoms of metabolic syndrome, compared with bacteria transferred from WT mice (Supplemental Figure 10, B–E). Conversely, we transferred WT and *Chst4^–/–^* ileal microbiota to *Chst4^–/–^* mice (Supplemental Figure 10F). As expected, this transfer rescued weight gain and symptoms of metabolic syndrome (Supplemental Figure 10, G–J). These findings indicate that the altered microbiota of *Chst4^–/–^* mice is sufficient for inducing obesity.

**Figure 7:**
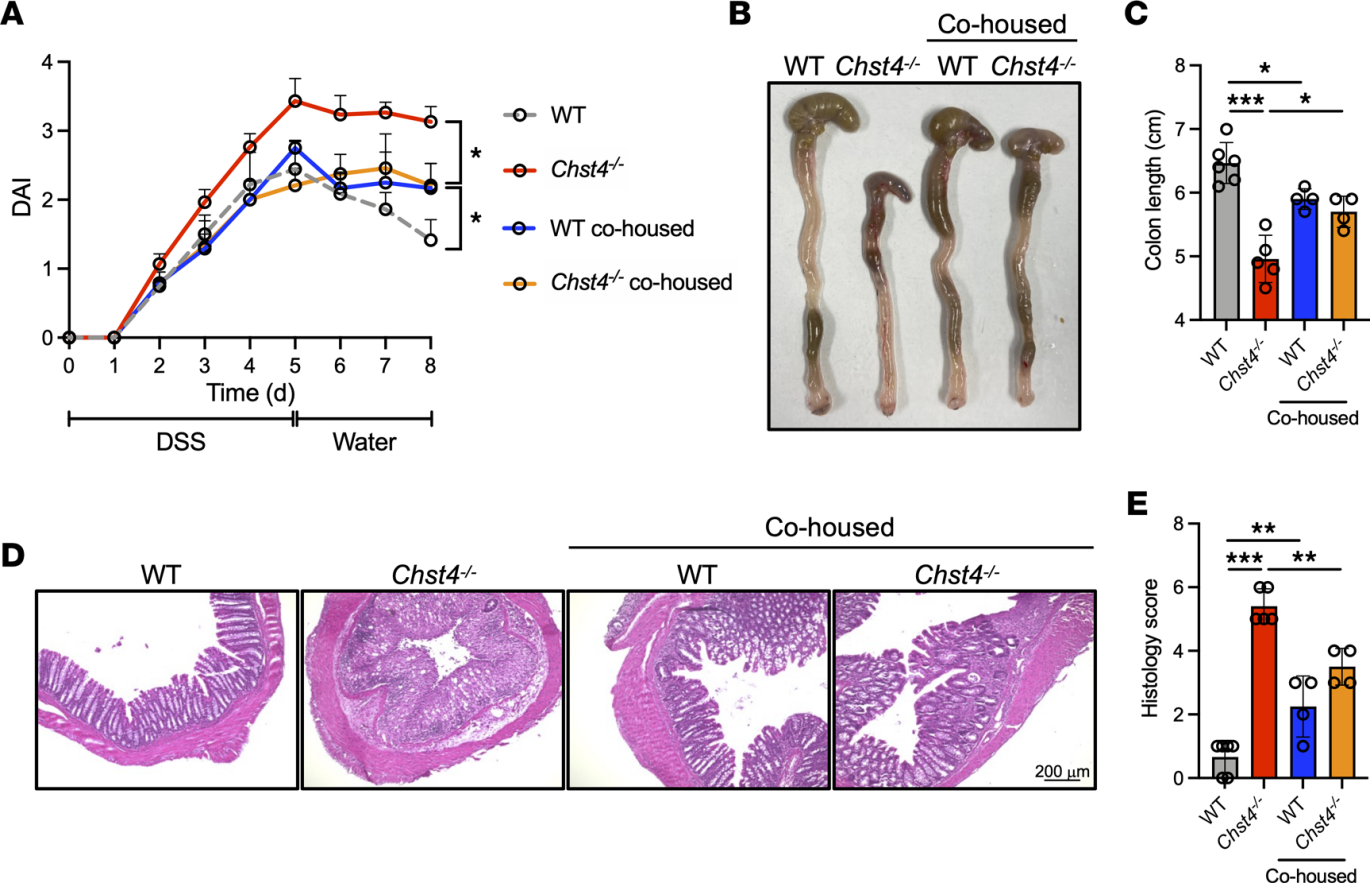
Modulation of gut microbiota rescues intestinal inflammation. (**A**) DAI score of mice treated with DSS for 5 days, followed by drinking water for 4 days. (**B**) Images of colons from WT and *Chst4^–/–^* mice. (**C**) Colon length of mice shown in **B**. (**D**) Representative image of H&E-stained colonic tissues. Scale bar: 200 μm. (**E**) Histology scores of mice shown in **D** (WT *n* = 6, WT/cohoused *n* = 4, *Chst4^–/–^* *n* = 5, *Chst4^–/–^*/cohoused *n* = 4). Data are representative of 2 independent experiments, and presented as the mean ± SD. *P < 0.05, **P <0.01, ***P < 0.001 via 2-way ANOVA followed by Tukey’s multiple-comparison test (**A**) or 1-way ANOVA (**C** and **E**).

We also investigated whether modulation of the gut microbiome could affect susceptibility to DSS-induced colitis. After 3 weeks of cohousing, mice were administered 3% DSS for 5 days following 4 days of regular water. Strikingly, cohoused *Chst4^–/–^* mice exhibited similar levels of weight loss and DAI score as cohoused WT mice (Figure 7A). Consistent with these findings, colon length and histology scores of WT and *Chst4^–/–^* mice were similar after cohousing (Figure 7, B–E). These data suggest a clear association between the altered bacteria composition observed in *Chst4^–/–^* mice and intestinal inflammation.

**Figure 8:**
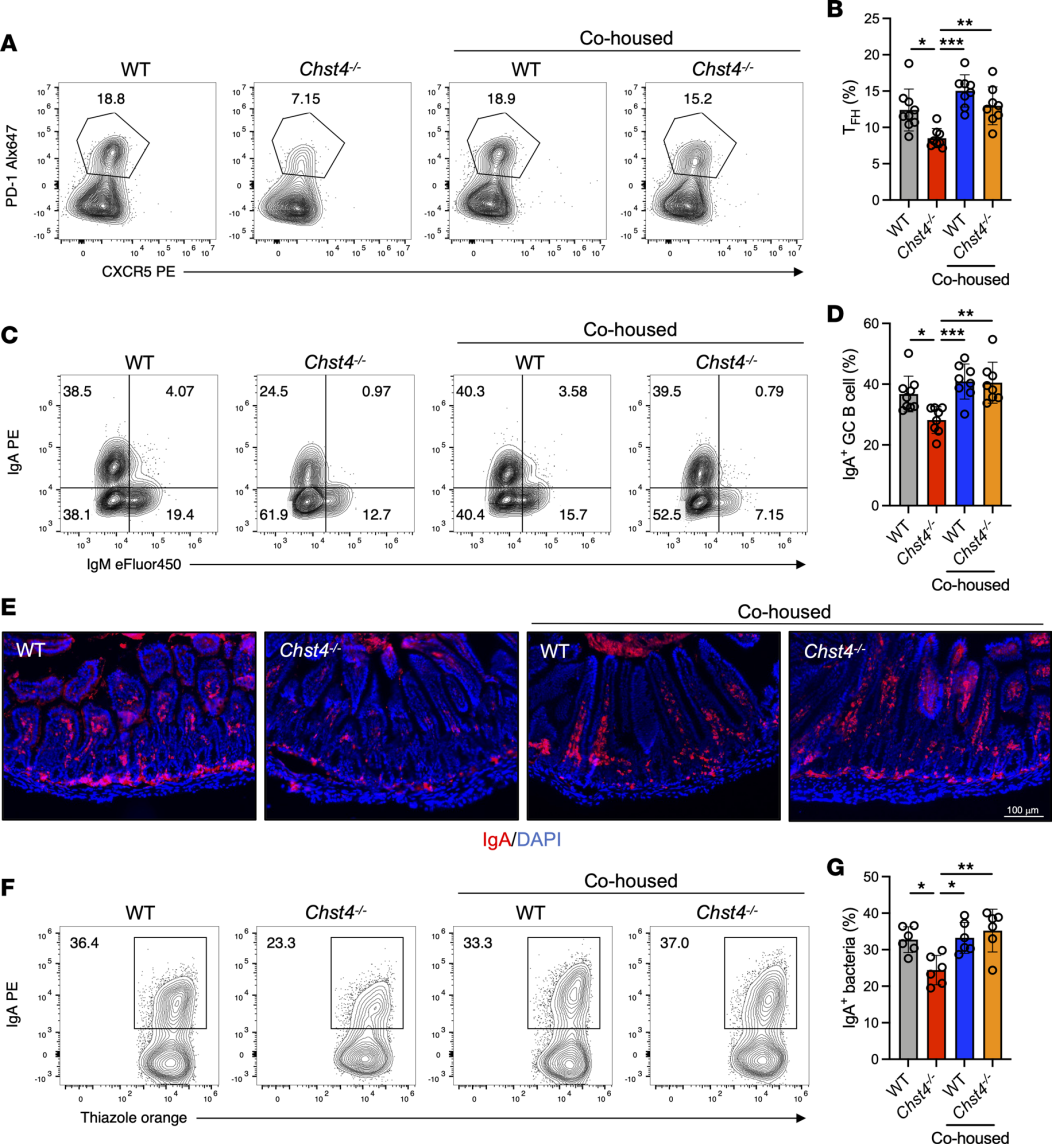
GlcNAc-6-O-sulfation on mucins enhances Tfh differentiation and IgA production via regulation of the gut microbiota. (**A**) Representative flow cytometry plot of Tfh cells from WT and *Chst4^–/–^* mice after 3 weeks of cohousing. (**B**) Frequency of Tfh cells shown in **A** (WT *n* = 9, WT/cohoused *n* = 8, *Chst4^–/–^* *n* = 8, *Chst4^–/–^*/cohoused *n* = 8). (**C**) Representative flow cytometry plot of GC B cells from WT and *Chst4^–/–^* mice. (**D**) Frequency of GC B cells in **C** expressing IgA (WT *n* = 9, WT/cohoused *n* = 8, *Chst4^–/–^* *n* = 8, *Chst4^–/–^*/cohoused *n* = 8). (**E**) Immunofluorescent staining of IgA in small intestinal tissue. Scale bar: 100 μm. (**F**) Representative flow cytometry plot of IgA-binding bacteria from WT and *Chst4^–/–^* mice (*n* = 6). (**G**) Frequency of IgA-binding bacteria in **F**. Data are representative of 2 independent experiments, and presented as the mean ± SD. *P < 0.05; **P < 0.01; ***P < 0.001 via 1-way ANOVA with Tukey’s multiple-comparison test.

Previous studies revealed that the microbiota regulate Tfh cell function, as indicated by the lower numbers of Tfh cells in germ-free mice (57). We hypothesized that the microbiota maintained via GlcNAc-6-O-sulfation on intestinal mucins positively regulate Tfh cells and GC B cells. As expected, the lower frequency of Tfh cells detected in *Chst4^–/–^* mice was recovered via cohousing with WT mice (Figure 8, A and B). Consistent with this finding, IgA-expressing GC B cells were increased after fecal microbiota modification by cohousing (Figure 8, C and D), as confirmed through IgA staining of small intestinal tissue (Figure 8E). Additionally, we found that the frequency of IgA-binding bacteria in *Chst4^–/–^* mice was restored upon cohousing (Figure 8, F and G). Altogether, these data indicate that the microbiota maintained by GlcNAc-6-O-sulfation of mucin O-glycan have a critical role in appropriate IgA responses.

## Discussion

We have previously reported that GlcNAc6ST-2, which is encoded by *Chst4*, predominantly catalyzes the GlcNAc-6-O-sulfation of O-glycans on mucins in the small and large intestine (12). The current data highlight a fundamental protective role of GlcNAc-6-O-sulfation for intestinal mucins against metabolic disease and intestinal inflammation. Previous studies have established that intestinal glycans are instrumental in host protection. Epithelial core 1– and core 3–derived O-glycan deficiency cause spontaneous colitis and reduced mucus thickness, indicating that O-glycans are essential for protection against intestinal inflammation (8–10). Furthermore, impaired mucosal O-glycosylation has been reported in patients with UC (58). While these findings demonstrated the significance of intestinal mucin O-glycosylation, the contribution of specific structures in complex glycans has remained unclear. In the absence of GlcNAc-6-O-sulfation of mucin O-glycans, the distance between bacteria and epithelial cells was reduced, and we detected an increase in the composition of *Bacteroides*, which are known members of mucin-degrading bacteria. This increase may have caused the mucin to degrade, promoting bacterial invasion into the mucus layer. Furthermore, several factors that define mucus stability have been reported including mucin viscosity, enzyme sensitivity, and cross linking (59, 60). These factors are capable of positively regulating mucin stability and protecting the host from bacteria, with a clear separation of the mucus layer. We do not exclude the contribution of sulfated O-glycans to these factors. Collectively, our findings provide strong evidence for the protective effect of GlcNAc-6-O-sulfation against obesity and intestinal inflammation. Additionally, our study using specific glycan structure–deficient mice suggests the concept that within complex structures of O-glycans, only limited structures such as sulfation play a significant role in biological function in vivo. This concept may hold true for other specific glycan structures, warranting further research into the matter.

The mechanisms underlying the selection of commensal bacteria and the colonization of beneficial bacteria in the gut are not yet fully understood. However, our study suggests that GlcNAc-6-O-sulfation of mucin glycans may be one of the mechanisms involved in the appropriate selection of bacteria. From our experiments, it is clear that the bacteria in *Chst4^–/–^* mice are the driving force for obesity and intestinal inflammation (Figure 6 and Supplemental Figure 10). Furthermore, our findings indicate that the susceptibility of *Chst4^–/–^* mice to HFD-induced obesity reemerged after they were separated following the cohousing period. This suggests that the transferred beneficial bacteria were unable to establish long-term colonization, and the composition of the gut microbiota reverted back to its original state (Supplemental Figure 9). In addition, we found that fecal bacteria composition in *Chst4^–/–^* mice partially shifted toward that of WT mice following the administration of WT mucins (Supplemental Figure 6). These data suggest that GlcNAc-6-O-sulfation is important for the selection and maintenance of beneficial gut microbiota. However, the incomplete recovery of the gut microbiota through mucin supplementation suggests the involvement of other factors, such as IgA (Supplemental Figure 6B). In this context, it is crucial to discuss the detailed mechanisms by which sulfation of mucin glycans contributes to the gut microbiota establishment. The utilization of mucin glycans by gut bacteria is vital for their survival, as evidenced by studies demonstrating that the deletion of the bacterial transcriptional factor ECF-σ, which normally activates mucin O-glycan utilization, results in a decline in microbial fitness within the gut (35). Similarly, host mucosal polysaccharides can be utilized by a wide range of bacterial strains that exhibit upregulated expression of carbohydrate-active enzymes in vivo (61). Sulfation has been proposed as a modification that regulates microbial colonization for a limited number of gut bacteria, as sulfatase deletion results in suppression of their growth in the gut (36). Sulfation of MUC2 glycans, which caps the terminal structure of the glycans, typically prevents bacterial degradation and utilization of oligosaccharides (62). Based on these studies and our findings, the loss of GlcNAc-6-O-sulfation might facilitate mucin glycan degradation, resulting in an increase in pathogenic bacteria that contribute to the weight gain of *Chst4^–/–^* mice. Further, a deficiency in 6-O-sulfation could potentially alter the adhesion of bacteria to the mucin layer (63). Collectively, our study strongly suggests that GlcNAc-6-O-sulfation on mucins is a key mechanism involved in the selection of commensal bacteria. This sulfation modification regulates the complex interactions between the host and gut microbiota, playing a crucial role in shaping the composition and dynamics of the microbial community in the gut.

In addition to a direct effect of sulfated glycans on the gut microbiota, we propose IgA-mediated regulation of the intestinal microbiome. Intestinal IgA has been shown to neutralize toxins and preferentially target colitogenic gut bacteria for clearance by M cells and intestinal macrophages (64). Further, the gut microbiota of individuals with IgA deficiency exhibits decreased richness and diversity (65). Additionally, previous adoptive transfer studies revealed that an intact Tfh cell/GC B cell/IgA axis is required for preventing obesity and DSS-induced colitis (44, 45). These studies demonstrate that IgA contributes to protection against obesity and intestinal inflammation via regulation of the gut microbiota. We detected an increased IgA production in feces from WT compared with *Chst4^–/–^* mice, which could positively regulate gut microbiota to protect against obesity and intestinal inflammation. Notably, given the dynamic and complex crosstalk between gut microbiota and the host immune system, more studies are needed to determine the degree of contribution of these 2 possible mechanisms to regulate intestinal microbiota.

Interestingly, our data strongly suggest regulation of the Tfh cell/GC B cell/IgA axis via gut microbiota and that dysregulation of this axis in *Chst4^–/–^* mice results in reduced IgA production. A previous study revealed that commensal microbiota–derived signals promote Tfh cell development, as germ-free mice have significantly lower Tfh cell numbers (57). Importantly, dysregulation of the Tfh cell/GC B cell/IgA axis in *Chst4^–/–^* mice could be rescued via microbiota modulation (Figure 8). Notably, the gut microbiota sustained via mucin sulfation seems to be sufficient to induce IgA class switching through the Tfh cell/GC B cell/IgA axis. While relatively little is known regarding the regulation of IgA, metabolites produced by commensal microorganisms, including short-chain fatty acids (SCFAs), have emerged as critical factors in this process (66–68). For example, acetate, a major metabolite produced by gut microbiota, has been shown to promote IgA production and direct selective IgA binding to certain bacteria via regulation of CD4^+^ T cells (69). Recent studies have highlighted the role of mucin glycans in microbiota modulation and the maintenance of mucosal homeostasis. O-glycans on intestinal mucins are utilized as an endogenous fermentation source by gut microbiota to produce SCFAs (70). Future studies employing transient colonization to identify the specific microbes regulating Tfh cell development and IgA production may help to shed light on how sulfated glycans on intestinal mucins regulate metabolic and immune homeostasis.

*Chst4* is expressed in intestinal epithelial cells as well as lymph node high endothelial venules (HEVs) (71). In HEVs, GlcNAc6ST-2 encoded by *Chst4* synthesizes 6-sulfo sialyl Lewis X, which is a ligand for L-selectin and plays a role in lymphocyte homing (72). We previously reported that *Chst4* is expressed in mesenteric lymph nodes but not in PPs (72). Moreover, *Chst4*-deficient mice showed a similar level of lymphocyte homing to PPs (72). Our data indicate that the defect in IgA production is restricted to PPs and the microbiota modulation by cohousing rescues phenotypes like weight gain and DSS-induced colitis susceptibility. These data strongly suggest a contribution of the gut microbiota rather than lymphocyte homing to the observed phenotype.

In conclusion, the present work highlights the role of intestinal sulfated glycans in complex interactions between the gut microbiome, metabolic regulation, and host immune system. First, we showed that GlcNAc-6-O-sulfation of mucin O-glycans has a protective role against obesity and intestinal inflammation. Second, we provided evidence that GlcNAc-6-O-sulfation regulates microbiota composition, which is implicated in obesity and intestinal inflammation. However, detailed mechanisms underlying how microbiota regulated by mucin O-glycan sulfation influences metabolism and intestinal inflammation remain to be elucidated. Enhancing sulfation to ameliorate inflammation and promote epithelial barrier function represents an attractive concept, which may apply not only to obesity and colitis, but also to other types of immune-mediated inflammatory diseases, such as nonalcoholic fatty liver disease and nonalcoholic steatohepatitis (73–75).

## Methods

### Mice.

C57BL/6 mice (Oriental Yeast) and *Chst4^–/–^* mice were maintained under the same conditions at Chiba University. *Chst4^–/–^* mice were generated as previously described (76). All animal experiments were performed using age- and sex-matched groups and were approved by the Chiba University Animal Care Committee.

### HFD treatment.

Mice housed in the facility were fed standard chow (Oriental Yeast). Age- and sex-matched male and female mice were used to compare weight gain under HFD feeding. Mice (7 weeks old) were fed an HFD of 32 kcal% (High Fat Diet 32, Clea Japan) for 6 weeks, and their body weight was monitored. For cohousing experiments, WT and *Chst4^–/–^* mice were housed in the same cage for 3 weeks before starting HFD and remained in cohousing for the duration of experiments. To conduct the cohousing experiment, we placed both WT and *Chst4^–/–^* mice together in the same cage for 3 weeks. Subsequently, we administered HFD to the mice for a period of 6 weeks. In the reseparation experiment, we initially cohoused the WT and *Chst4^–/–^* mice for 3 weeks. Following this cohousing period, we separated the mice and continued to house them separately for an additional 3 weeks. After the separation period, the mice were then fed HFD for 6 weeks.

### Antibiotics treatment.

WT and *Chst4^–/–^* mice were administered drinking water containing 0.5 mg/mL ampicillin (A9518-25G, Sigma-Aldrich), neomycin (24129-42, Nacalai Tesque), and vancomycin (226-01306, FUJIFILM) for 6 weeks while fed an HFD to determine the contribution of microbiota to weight gain.

### Fasting glucose test.

Mice were fasted for 15 hours prior to measurement of blood glucose concentration. Glucose levels were detected using a Medisafe FIT Blood Glucose Meter (MS-FR201B, TERUMO) and Medisafe FIT Blood Glucose Tip (MS-FC030, TERUMO).

### Glucose tolerance test.

Mice were fasted for 4 hours prior to glucose challenge. Glucose levels were detected as described above. Glucose (2 mg/g body weight; 047-31161, FUJIFILM) was injected intraperitoneally, and blood glucose concentration was measured at 15, 30, 60, 120, and 180 minutes.

### Insulin resistance test.

Mice were fasted for 4 hours prior to insulin challenge. Glucose levels were detected as described above. Insulin (0.75 U/kg body weight; 12878-86, Nacalai Tesque) was injected intraperitoneally, and blood glucose concentration was measured at 15, 30, 60, and 120 minutes.

### RNA isolation and qPCR.

Total RNA was isolated using TRIzol reagent (15596026, Invitrogen) as per the manufacturer’s protocol. cDNA was generated using the ReverTra Ace qPCR RT Master Mix with gDNA Remover kit (FSQ-301, TOYOBO). qPCR was performed with SYBR Green on a CFX96 Touch Real Time PCR Detection System (Bio-Rad Laboratories). Gene expression was normalized to that of *Actb*. Primers used were *Actb* Fw CATCCGTAAAGACCTCTATGCCAAC and Rv ATGGAGCCACCGATCCACA; *Tnfa* Fw CTACTCCCAGGTTCTCTTCAA and Rv GCAGAGAGGAGGTTGACTTTC; *Il1b* Fw ACAGGCTCCGAGATGAACAA and Rv GGCCACAGGTATTTTGTCGT; *Il6* Fw CCTGAGACTCAAGCAGAAATGG and Rv AGAAGGAAGGTCGGCTTCAGT; *Ifng* Fw CAATCAGGCCATCAGCAACAA and Rv GACTCCTTTTCCGCTTCCTGA; *Il17a* Fw CAGGGAGAGAGCTTCATCTGTGT and Rv GCTGAGCTTTGAGGGATGAT; *Cxcl1* Fw TGGCTGGGATTCACCTCAAG and Rv TCTCCGTTACTTGGGGACAC; *Cxcl2* Fw CTCTCAAGGGCGGTCAAAAAG and Rv GAGGCACATCAGGTACGATCC; *Cxcl5* Fw CCCTACGGTGGAAGTCATAGC and Rv TTCACTGGGGTCAGAGTCCT; *Ccl2* Fw CTGGAGCATCCACGTGTTGG and Rv CATTCCTTCTTGGGGTCAGCA; *Arg1* Fw AACACTCCCCTGACAACCAG and Rv TCTACGTCTCGCAAGCCAAT; *Nos2* Fw GCCTTGCATCCTCATTGGGC and Rv GGAACAGCACTCTCTTGCGG; *Cybb* Fw GCCAGTGTGTCGAAATCTGC and Rv TGGCATTCACACACCACTCA; *Lcn2* Fw GTCCCCACCGACCAATGC and Rv GGGGAGTGCTGGCCAAATAA; *Mpo* Fw GCTGGAGAGTCGTGTTGGAA and Rv GAGCAGGCAAATCCAGTCCT; *Il21* Fw CGCCTCCTGATTAGACTTCGT and Rv GGCTTGAGTTTGGCCTTCTG; and *Il10* Fw AGTACAGCCGGGAAGACAAT and Rv AAGGCTTGGCAACCCAAGTAA.

### DSS-induced colitis.

Mice were administered 2% (w/v) DSS (160110, MP Biomedicals) via drinking water for 5 days and then switched to normal drinking water. Mice were monitored daily for signs of disease with a score of 0–4 assigned for weight loss, stool consistency, and presence of blood in the stool. The individual scores were added, and the average score was DAI.

### CAC model.

Mice were injected with AOM (10 mg/kg, 011-20171, FUJIFILM) diluted in PBS and then subjected to 3 cycles of DSS treatment, with each cycle consisting of 1.5% DSS for 5 days, followed by 15 days with normal water. After protocol completion, colonic tumors were counted. Tumor surface area was measured using ImageJ software (NIH).

### Immunofluorescent staining.

For immunofluorescent staining, tissue sections were permeabilized with 0.3% Triton X-100 and blocked in 3% bovine serum albumin (BSA) for 1 hour at room temperature prior to incubation overnight with an anti–Ki-67 antibody (ab15580, Abcam), anti–mouse Gr-1 antibody (108401, BioLegend), or an anti–mouse IgA antibody (A90-103A, Bethyl Laboratories) at 4°C. Alexa Fluor 488–conjugated anti–rabbit IgG secondary antibody (A21206, Invitrogen) or Alexa Fluor 647–conjugated anti–rat IgG secondary antibody (A21247, Invitrogen) was added for 1 hour. Images of sections were obtained using a BZ9000 microscope (KEYENCE).

### DNA isolation and 16S rRNA sequencing of ileal and fecal bacteria.

For gut microbiota analysis, we utilized 2 cages of WT and *Chst4^–/–^* mice to distinguish between differences in composition owing to cage-cluster effects and genotype. Ileal and fecal bacteria DNA was isolated using NucleoSpin DNA Stool (U0472B, Macherey-Nagel). The 16S rRNA gene V3 and V4 regions were amplified via PCR, and sequence processing was performed using paired-end MiSeq with 250-bp reads. Sequence data analysis was performed using Quantitative Insights Into Microbial Ecology 2 (QIIME2, version 2021.4.0; https://qiime2.org). Forward and reverse sequence merging, quality control, and ASV clustering were performed using DADA2 (https://benjjneb.github.io/dada2/). Taxonomic classification was performed using the Silva 132 ASV full-length sequence database (https://www.arb-silva.de). The phylogenic tree was constructed via FastTree and used for computing the unweighted UniFrac distances between samples. A PCoA plot was used to assess the variation between experimental groups (β diversity). Alpha diversity curves were generated using the Chao1 index. LEfSe analysis was used to investigate bacterial members that drive differences between groups.

### Isolation of lamina propria cells from the small and large intestine.

Small or large intestinal tissues were cut into 0.5-cm pieces and transferred into 50 mL conical tubes. The tubes were then shaken at 200 rpm for 20 minutes at 37°C in Hanks’ balanced salt solution (HBSS) (084-08345, FUJIFILM) supplemented with 5% fetal bovine serum (FBS) and 5 mM EDTA. This process was repeated twice. Cell suspensions were passed through a cell strainer, and the remaining intestinal tissues were washed and minced, transferred to 50 mL conical tubes, and shaken for 30 minutes at 37°C in HBSS supplemented with 5% FBS and collagenase solution (1 mg/mL; 034-2236, FUJIFILM). Cell suspensions were passed through a cell strainer and pelleted via centrifugation at 800*g*. Percoll (17089101, Cytiva) gradients (40%–80%) were performed for isolating lamina propria mononuclear cells.

### Microbiota transplantation.

Fresh ileal contents from WT or *Chst4^–/–^* mice were collected and suspended in sterile PBS at 200 mg/mL concentration. WT or *Chst4^–/–^* mice were treated with an antibiotic cocktail containing 0.5 mg/mL ampicillin (A9518-25G, Sigma-Aldrich), neomycin (24129-42, Nacalai Tesque), vancomycin (226-01306, FUJIFILM), and metronidazole (130-18062, FUJIFILM) for 2 weeks via drinking water, followed by oral gavage with 200 mL of ileal contents. Transplantation was performed once. One week later, all recipients started receiving HFD feeding, and weight gain was monitored for 6 weeks.

### Flow cytometry.

Fluorescently labeled antibodies specific for CD4 (RM4-5, 100509), CD11b (M1/70, 101211), CD19 (1D3, 152403), CD25 (PC61, 102051), CD44 (IM7, 103029), CD45 (30-F11, 103107, 103115, 103149), CD138 (281-2, 142505), PD-1 (RMP1-30, 109117), CXCR5 (SPRCL5, 12-7185-82), B220 (RA3-6B2), Bcl6 (7D1, 358511), GL7 (GL7, 144619), Fas (SA367H8, 152603), IgA (mA-6E1, 12-4204-82), IgM (eB121-15F9, 48-5890-82), IgG1 (RMG1-1, 406619), IgG2b (RMG2b-1, 406707), Ly6G (1A8, 127607), and Ly6C (HK1.4, 128007) were purchased from eBioscience and BioLegend. Fc block (2.4G2, 70-0161-U500) was purchased from TOMBO Bioscience. Dead cells were stained using the Zombie Aqua or Zombie NIR fixable dye staining kit (423101 or 423105, BioLegend). Intracellular staining was performed using an Intracellular Fixation and Permeabilization Buffer Set (00-5523-00, Invitrogen). For the analysis of IgA-coated fecal bacteria, feces were collected in a 1.5 mL tube, suspended in cold PBS, and then centrifuged at 50*g* to remove debris. Supernatants were then placed in new 1.5 mL tubes and washed twice with cold PBS via centrifugation at 8,000*g*. Bacterial pellets were blocked with 200 μL PBS containing 3% (w/v) BSA for 1 hour on ice. Without washing, bacteria were stained with an anti–mouse IgA conjugated to PE (mA-6E1, 12-4204-82, eBioscience) for 1 hour on ice. The pellet was washed twice via centrifugation at 8,000*g* for 5 minutes and then stained with thiazole orange for 1 hour on ice. Finally, bacteria were suspended in 200 μL PBS and analyzed using flow cytometry. Flow cytometric analysis was performed on a Beckman Coulter Cytoflex flow cytometer and analyzed using FlowJo software (BD Biosciences).

### Quantification of fecal IgA, IgG1, and IgG2b.

To quantify fecal IgA, IgG1, and IgG2b, feces were collected in 1.5 mL tubes and stored at –80°C until experiments. Fecal pellets (1 mg) were homogenized in 1 mL cold PBS. Following centrifugation at 16,000*g* for 10 minutes at 4°C, the supernatant was analyzed by ELISA. Antibody concentration was measured using sandwich ELISA. Plates (96-well) were coated with 1 μg/mL capture antibody (anti–mouse IgA, A90-103A, Bethyl Laboratories; anti-mouse IgG, 405303, BioLegend) overnight at 4°C. Plates were washed and blocked with 3% BSA in PBS for 1 hour at room temperature. Samples and standard were incubated for 2 hours at room temperature. Captured antibody was detected using a horseradish peroxidase–conjugated antibody (IgA detection, 1040-05, SouthernBiotech; IgG1 detection, 1070-05, SouthernBiotech; IgG2b detection, 1070-05, SouthernBiotech;). The color reaction was developed using TMB substrate and terminated using 2 M H_2_SO_4_.

### Oil Red O staining.

Liver tissues were embedded in OCT compound (4583, SAKURA Tissue-Tek OCT Compound) and cut into 7-μm-thick sections. Oil Red O (O0625-25G, Sigma-Aldrich) was dissolved in 0.5% (w/v) isopropanol as stock solution. The stock solution was filtered, and distilled water was added to 0.3% Oil Red O. Tissue slides were fixed with 10% formalin for 5 minutes and stained with fresh 0.3% (w/v) Oil Red O solution. After staining, slides were stained with hematoxylin for nuclear staining.

### Alcian blue staining and high-iron diamine–Alcian blue staining.

For Alcian blue staining, paraffin-embedded blocks were sectioned to 6 mm thickness. Tissue slides were stained with Alcian blue solution (pH 1.0) (1.01647.0500, Sigma-Aldrich) and counterstained with Nuclear Fast Red (N0184, Tokyo Chemical Industry). High-iron diamine–Alcian blue staining for sulfated mucins was performed as previously described (11, 77). Tissue slides were incubated for 16 hours in solution containing *N*,*N*-dimethyl-*para*-phenylenediamine (D3931, Tokyo Chemical Industry), *N*,*N*-dimethyl-*meta*-phenylenediamine (219223-5G, Sigma-Aldrich), and ferric chloride (157740-100G, Sigma-Aldrich), rinsed in dH_2_O, and stained with Alcian blue pH 2.5 (1.01647.0500, Sigma-Aldrich).

### Administration of mucins isolated from large intestine.

Mucin isolation from colonic tissues was performed as previously described (11). Colons from WT mice were opened longitudinally and luminal contents were carefully removed. Adherent mucins were gently scraped off with a glass slide. Collected mucins were suspended in 1 mL guanidium chloride (GuCl) extraction buffer (6 M GuCl, 0.1 M Tris pH 8.0, 1 mM EDTA) containing cOmplete Protease Inhibitors (11836153001, Roche) and dispersed with a Dounce homogenizer. Samples were then extracted overnight at 4°C on a rotator. Then, samples were pelleted and suspended in extraction buffer and re-extracted for 4 hours. Following re-extraction, samples were reduced twice with 100 mM dithiothreitol (042-29222, FUJIFILM), once for overnight at 37°C, and the other for 4 hours at 37°C to solubilize the mucin. Mucins were then alkylated with 250 mM iodoacetamide on a rotator overnight at 25°C in the dark. The reduced and alkylated mucins were dialyzed into PBS and postdialyzed samples were concentrated by Amicon Ultra-4 Centrifugal Filters (100 kDa MWCO, UFC810024, Millipore). The concentrated samples were stored at –80°C until administration. Isolated mucins were validated by SDS-PAGE using 5%–12 % gradient gels (199-15191, FUJIFILM). Gels were stained with PAS (1.01646.0001, Sigma-Aldrich) and Alucian blue (pH 1.0) (1.01647.0500, Sigma-ALdrich) following the manufacturer’s protocols. Mucin solutions were administered via gastric gavage every other day over the course of 2 weeks, after which feces were collected and bacterial composition was analyzed using 16S rRNA sequencing.

### H&E staining.

For H&E staining, tissue was fixed with 10% formalin overnight at 4°C before paraffin embedding. Tissues were cut into 7-μm-thick sections and stained with hematoxylin (131-09665, FUJIFILM) and eosin (050-06041, FUJIFILM).

### Carnoy’s fixation and staining of the mucus layer.

Colonic tissues were placed in Carnoy’s fixative solution (60% methanol, 30% chloroform, 10% glacial acetic acid) for 1 week. After paraffin embedding, tissues were then washed with ethanol for 30 minutes and xylene for 30 minutes, 3 times each. Paraffin-embedded blocks were sectioned to 7 mm thickness. The hybridization step was performed at 50°C overnight with an EUB338 probe (5 mg/mL, MBD0033-50UL, Sigma-Aldrich) in hybridization buffer (20 mM Tris HCl, 50 mM NaCl, 0.1% SDS). After washing with PBS, 3% BSA–containing blocking solution was added for 30 minutes at room temperature. Anti-MUC2 primary antibody (H-300, sc-15334, Santa Cruz Biotechnology) was added overnight at 4°C. After washing with PBS, an Alexa Fluor 488–conjugated anti–rabbit IgG secondary antibody (A21206, Invitrogen) and DAPI were applied for 1 hour at room temperature. After washing, slides were mounted using Fluoromount mounting media (K024, Diagnostic BioSystems). To stain the b1 and b2 layers of the colon sections, we initially incubated them with the EUB338 probe. Subsequently, the sections were stained using an anti-MUC2 primary antibody and biotinylated MALII lectin (B-1265-1, Vector Laboratories). For visualization, an Alexa Fluor 488–conjugated anti–rabbit IgG secondary antibody was applied, along with Streptavidin–DyLight 405 (016-470-084, Jackson ImmunoResearch) and DAPI, for 1 hour at room temperature. Images of sections were obtained using a BZ9000 microscope (KEYENCE). Mucus thickness was measured by NIH ImageJ/Fiji software.

### Organoid culture.

Isolation of crypt cells and organoid cultures was performed as previously described (16). Large intestine organoids were established from freshly isolated tissue. Isolated large intestines were cut longitudinally and washed with cold PBS. Colonic tissues were incubated in Gentle Cell Dissociation Reagent (100-0485, STEMCELL Technologies) for 50 minutes at room temperature and crypts were released from tissues by pipetting. Crypts were then passed through a 70-μm cell strainer, and the crypt fraction was enriched via centrifugation. Crypts were subsequently embedded in Matrigel (356234, Corning) and plated. After polymerization of Matrigel, culture media (06005, STEMCELL Technologies) was added and refreshed every 3 days. After 5–6 days in culture, total organoid numbers per well were counted to evaluate organoid efficiency. Budding and surface area were calculated by NIH ImageJ/Fiji software as previously described (78).

### Staining of human colonic tissue.

Archived formalin-fixed, paraffin-embedded specimens obtained from patients with UC and colonic adenoma at North Medical Center, Kyoto Prefectural University of Medicine were used. For controls, we used histologically normal nontumor areas of endoscopically resected specimens. Two consecutive sections (4 mm thick) were used for Alcian blue pH 1.0 staining and MUC2 immunostaining. For Alcian blue pH 1.0 staining, after briefly rinsing with 0.1N HCl, sections were stained using 1% Alcian blue solution, pH 1.0 (Muto Pure Chemicals) for 30 minutes followed by another brief rinse with 0.1N HCl. For MUC2 staining, immunohistochemistry was performed by SRL, Inc. using a purified mouse anti–human MUC2 antibody (CCP58, 555926, BD Pharmingen) followed by visualization with DAB. Images of the same region from 2 serial sections (randomly selected ×100 area) were obtained using NIS-Elements (NIKON) and analyzed by NIH ImageJ/Fiji software to measure the values of integrated density. Then, each value from Alcian blue pH 1.0 staining was divided by the corresponding MUC2 staining value to obtain the Alcian blue pH 1.0/MUC2 ratio.

### Statistics.

All statistical analyses were performed with GraphPad Prism software, version 9. One-way or 2-way ANOVA followed by Tukey’s multiple-comparison test, or 2-tailed independent Student’s *t* test was used to determine significance. A *P* value of less than 0.05 was considered significant (*P < 0.05, **P < 0.01, ***P < 0.001).

### Study approval.

All animal experiments were approved by the Chiba University Animal Care Committee. For staining of human colonic tissue, informed consent was obtained, and the protocol was approved by the Ethics Committee of the Kyoto Prefectural University of Medicine (protocol no. ERB-C-1408-2).

### Data availability.

All data are available in the main text or supplementary material. Values for all data points are provided as a supplemental data value file. Raw Illumina paired-end sequence data are available in the DNA Data Bank of Japan under accession number PRJDB16080.

## Author contributions

HA and HK conceived the idea for this project and designed the experiments. HA performed most experiments and analyzed data. HA analyzed the 16S sequencing data. AH and TI performed pathological analysis. AM, MS, and KT provided technical support. TLD provided critical discussion. The manuscript was written by HA and HK.

## Supplementary Material

Supplemental data

Supporting data values

## Version 1 07/18/2023

In-Press Preview

## Version 2 08/22/2023

Electronic publication

## Version 3 08/31/2023

N-acetylglucosamine-6-O sulfation and N-acetylglucosamine (GlcNAc)-6-O sulfation were changed to include a hyphen before sulfation
