# Supplementary material for: N-acetylglucosamine-6-O-sulfation on intestinal mucins prevents obesity and intestinal inflammation by regulating gut microbiota
Source: JCI Insight. 2023 Aug 22;8(16):e165944. doi: 10.1172/jci.insight.165944 (PMC10543739; doi:10.1172/jci.insight.165944)
Supplement: Supplemental data [file jciinsight-8-165944-s270.pdf]

Supplemental Figure

***N*-acetylglucosamine-6-*O*-sulfation on intestinal mucins prevents obesity and intestinal inflammation by regulating gut microbiota**

Abo et al.

This PDF file includes supplemental figure 1 to 10

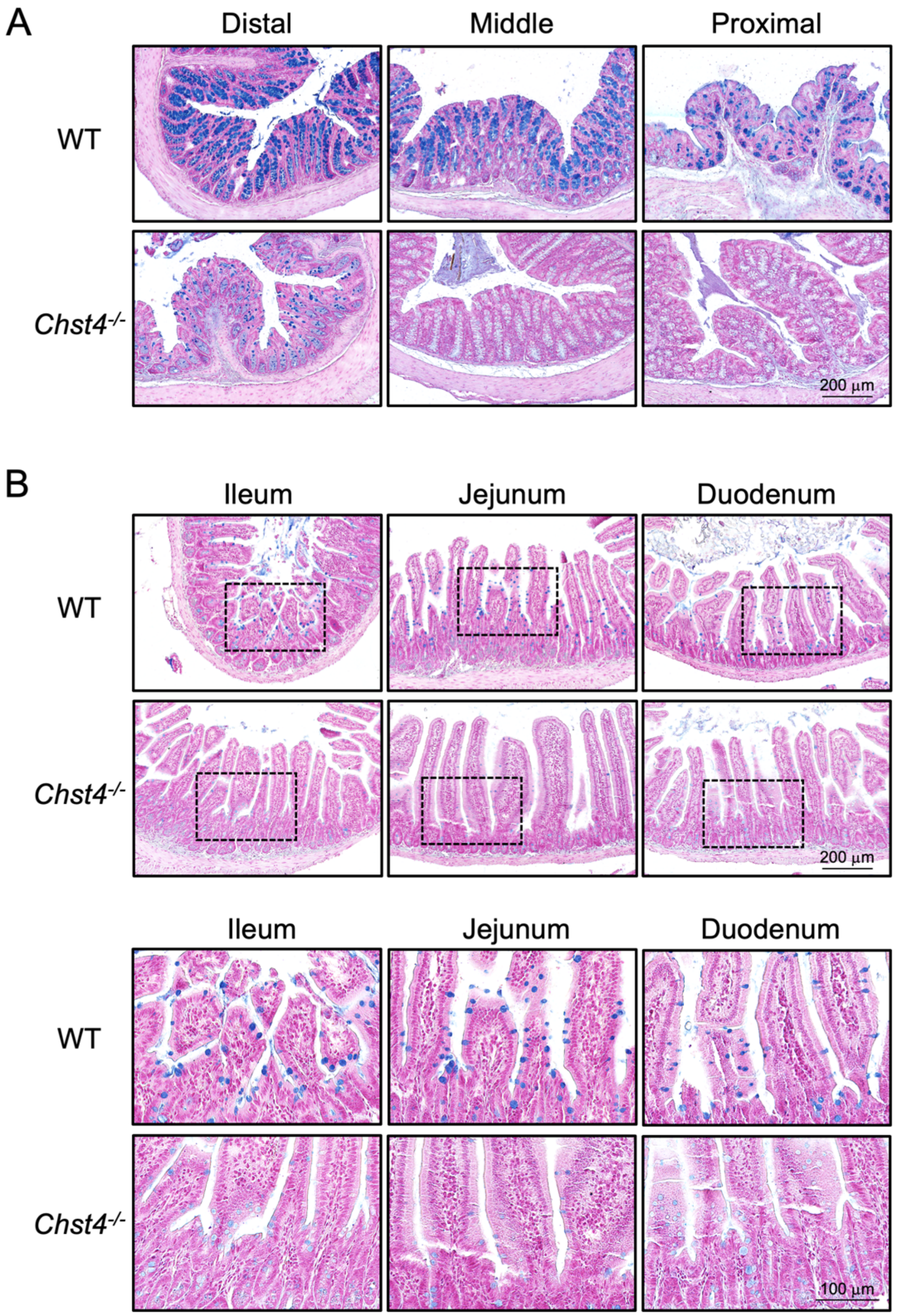

Supplemental Fig. 1. Deletion of *Chst4* results in a remarkable loss of sulfation in intestinal tissues as confirmed by Alcian blue staining.

(A) Alcian blue staining (pH 1.0) of the large intestine in WT and *Chst4*$^{-/-}$ mice. **(B)** Alcian blue staining (pH 1.0) of the small intestine in WT and *Chst4*$^{-/-}$ mice. Top panel; low magnification, bottom panel; high magnification.

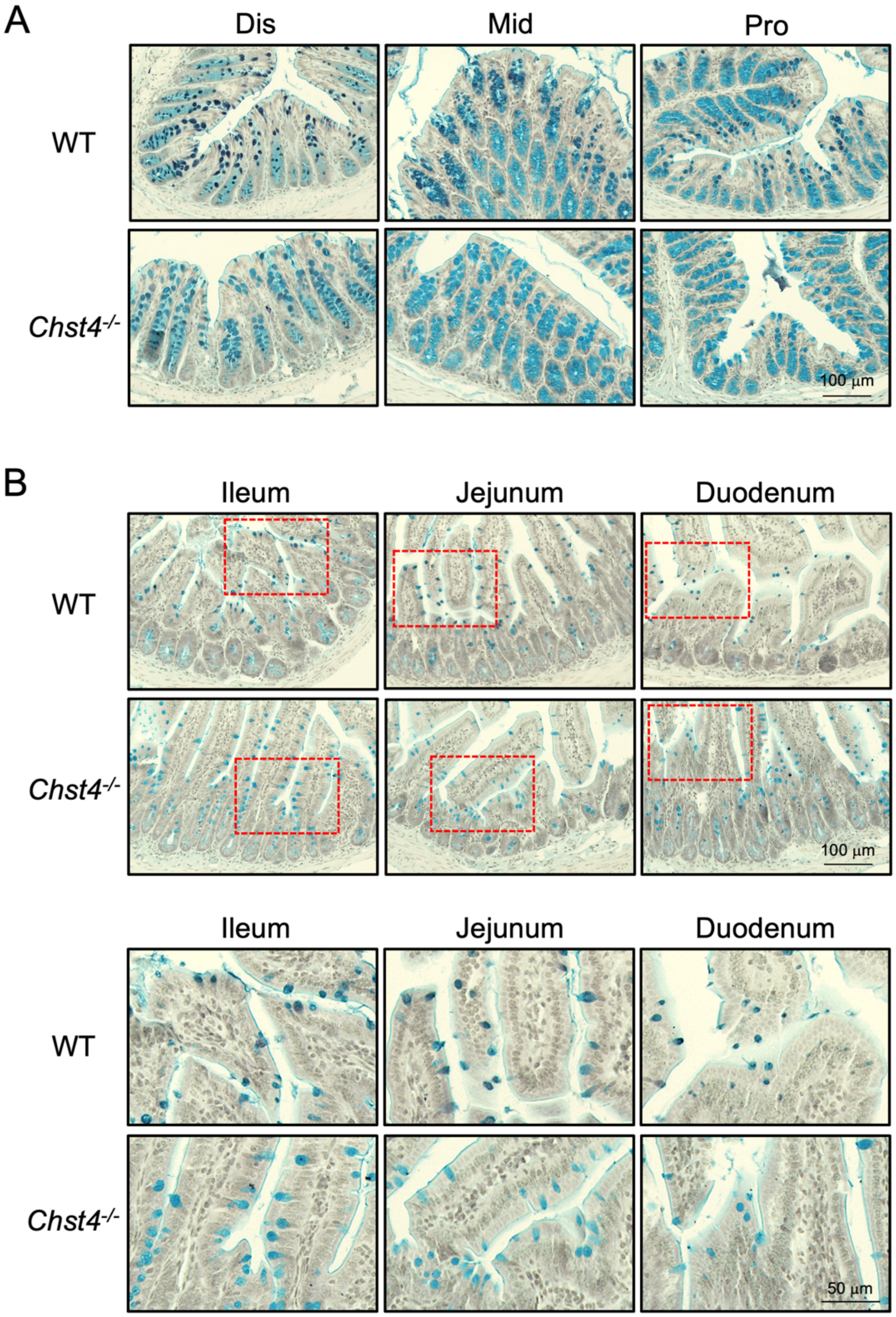

Supplemental Fig. 2. Deletion of *Chst4* leads significant loss of sulfation in intestinal tissues as confirmed by HID-AB staining.

(A) HID-AB staining (pH 2.5) of the large intestine in WT and *Chst4*$^{-/-}$ mice. **(B)** HID-AB staining (pH 2.5) of the small intestine in WT and *Chst4*$^{-/-}$ mice. Top panel; low magnification, bottom panel; high magnification.

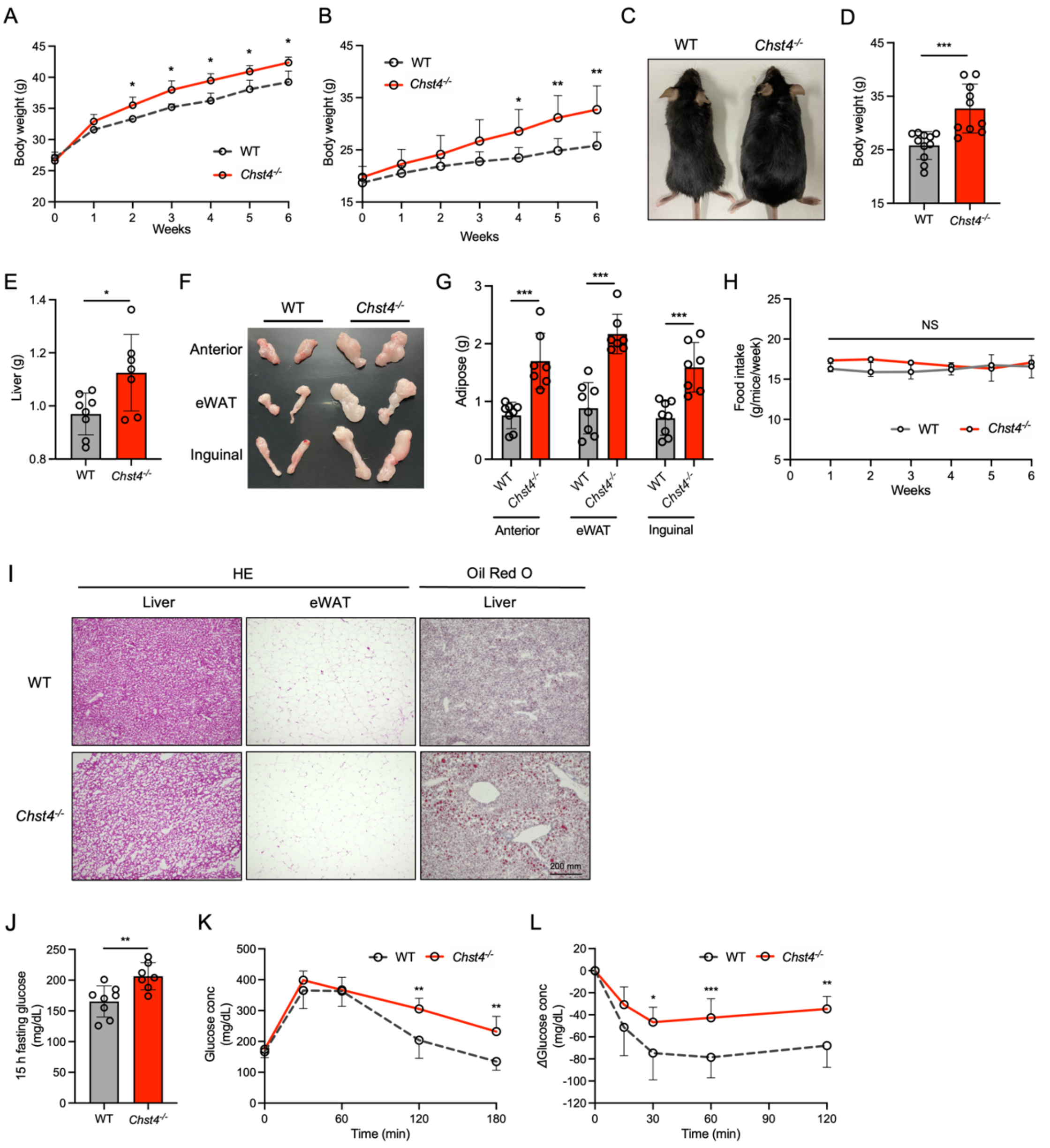

Supplemental Fig. 3. *Chst4$^{-/-}$* mice develop HFD-induced obesity.

(A) Weight gain of male WT and *Chst4$^{-/-}$* mice over time (n = 3). **(B)** Weight gain in female WT and *Chst4$^{-/-}$* mice fed an HFD for 6 weeks. **(C)** Representative images of WT and *Chst4$^{-/-}$* mice after 6 weeks feeding of HFD. **(D)** Total weight of WT and *Chst4$^{-/-}$* mice fed an HFD (WT n = 11, *Chst4$^{-/-}$* n = 10). **(E)** Liver weight in WT and *Chst4$^{-/-}$* mice. **(F)** Representative image of the adipose tissue. **(G)** Adipose tissues weight of WT and *Chst4$^{-/-}$* mice (WT n = 8, *Chst4$^{-/-}$* n = 7). **(H)** Food intake was measured based on the weight of the HFD (n = 4). **(I)** Representative images of the liver and adipose tissues stained with HE and Oil Red O in WT and *Chst4$^{-/-}$* mice. **(J)** 15-h fasting blood glucose concentration after 6 weeks of HFD feeding. **(K)** Blood glucose concentration in glucose tolerance tests. **(L)** Change in blood glucose concentration in WT and *Chst4$^{-/-}$* mice after

additional insulin challenge following 6 weeks of HFD feeding (WT n = 8, *Chst4*$^{-/-}$ n = 7). Data are representative of two independent experiments, and presented as the mean ± SD; **P* < 0.05, ***P* < 0.01, ****P* < 0.001 via unpaired, two-tailed *t*-tests or two-way ANOVA.

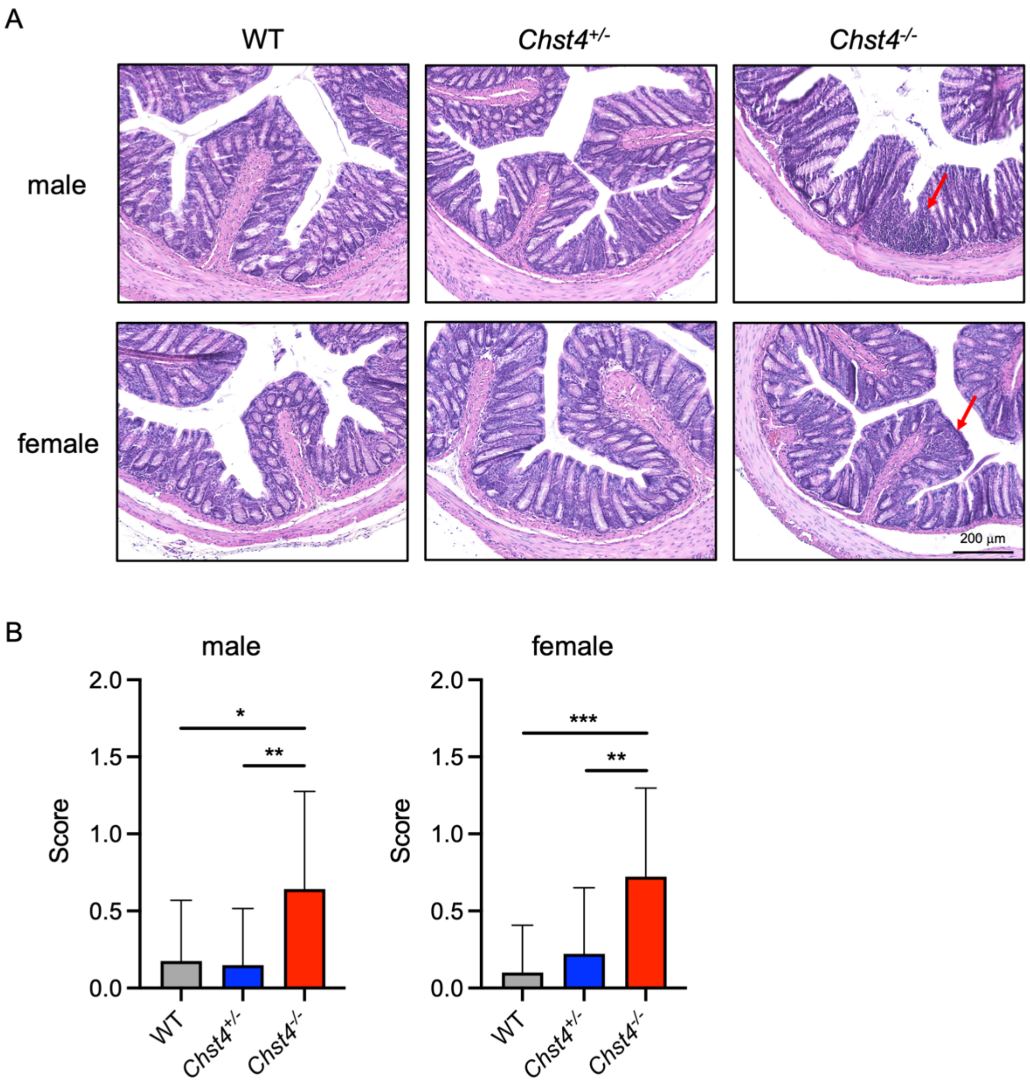

Supplemental Fig. 4. *Chst4$^{-/-}$* mice develop low grade inflammation.

(A) Representative images of colon tissues from littermate control. **(B)** Histology score shown in A (male; WT n = 17, *Chst4$^{+/-}$* n = 20, *Chst4$^{-/-}$* n = 14, female; WT n = 20, *Chst4$^{+/-}$* n = 18, *Chst4$^{-/-}$* n = 18). Data are presented as the mean ± SD; *P < 0.05, **P < 0.01, ***P < 0.001 via one-way ANOVA with multiple comparison tests.

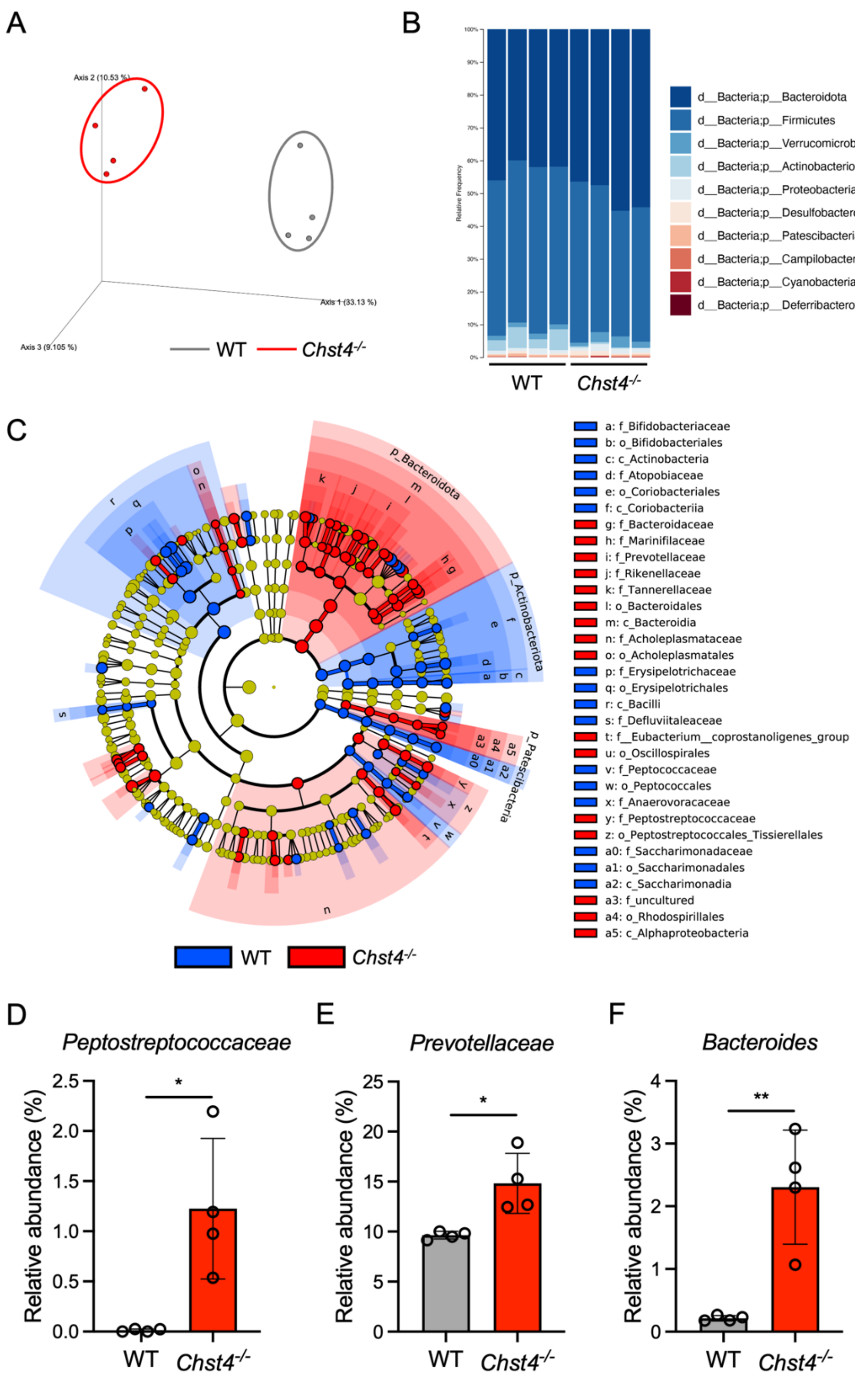

Supplemental Fig. 5. Loss of mucin *O*-glycan sulfation alters fecal microbiota composition. **(A)** Principal coordinates analysis of fecal microbiota based on unweighted UniFrac distances. **(B)** Relative abundance of bacteria at the phylum level. **(C)** LEfSe analysis of colonic microbiota taxa significantly different between WT and *Chst4*$^{-/-}$ mice (n = 4). **(D-F)** Relative abundance of indicated bacteria in fecal samples from WT and *Chst4*$^{-/-}$ mice. Data are presented as the mean ± SD; **P* < 0.05, ***P* < 0.01 via unpaired, two-tailed *t*-tests.

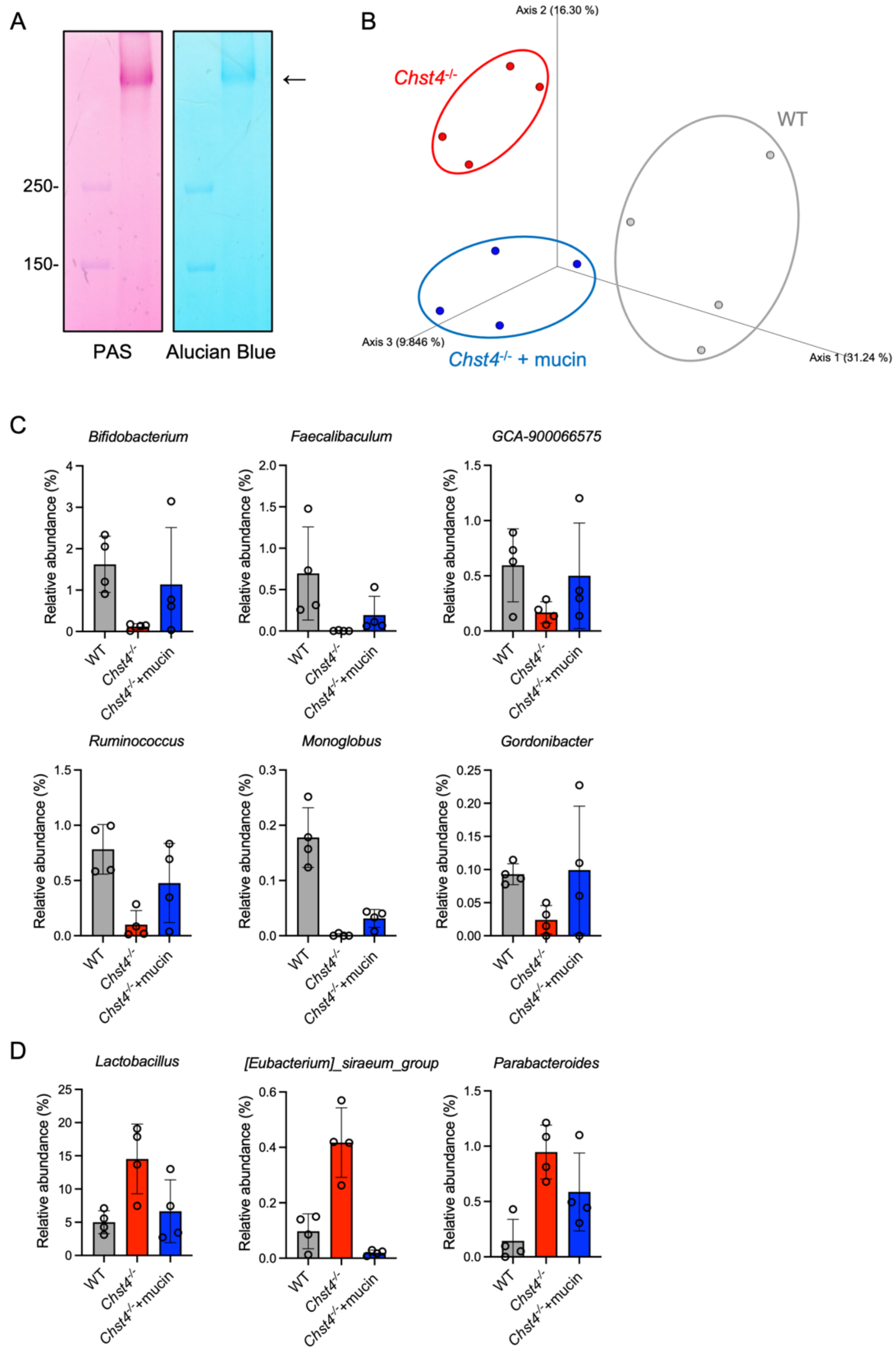

Supplemental Fig. 6. Mucin administration alters bacteria composition in *Chst4*$^{-/-}$ mice.

(A) PAS and Alcian blue staining (pH. 1.0) following SDS-PAGE. **(B)** Principal coordinates analysis of fecal microbiota based on unweighted UniFrac distances. **(C)** Bacteria genus that were increased by mucin administration. **(D)** Bacteria genus that were decreased by mucin administration.

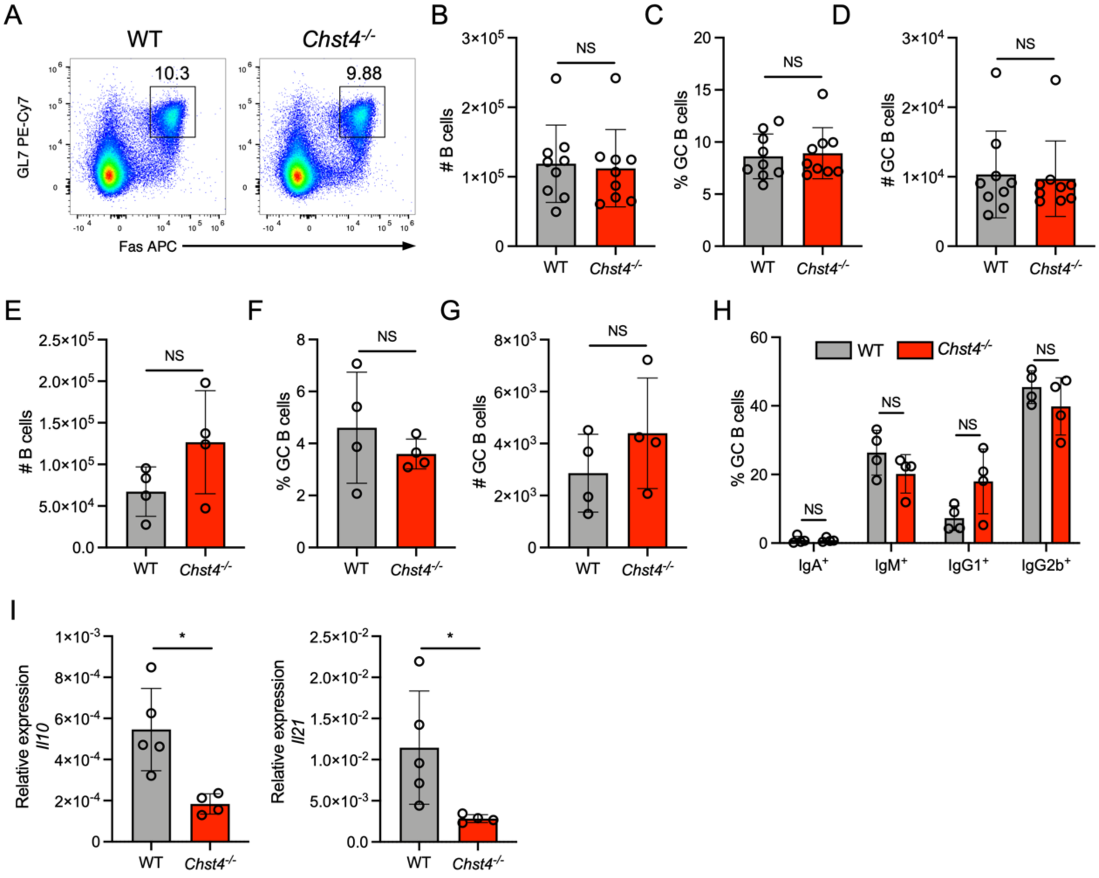

Supplemental Fig. 7. B cell subsets in PPs and mLNs and expression of cytokines related to IgA production.

(A) Representative FACS plot for GC B cells defined as $CD45^+CD19^+GL7^+Fas^+$ in PPs from WT and *Chst4*$^{-/-}$ mice. **(B)** Absolute cell number of total B cells in PPs defined as $CD45^+CD19^+$. **(C-D)** Frequency and cell number of GC B cells shown in a (n = 9). **(E)** Absolute cell number of total B cells defined as $CD45^+CD19^+$ in mLNs. **(F-G)** Frequency and cell number of GC B cells in mLNs. **(H)** Frequency of IgA^+, IgM^+, $IgG1^+$, and $IgG2b^+$ GC B cells in mLNs (n = 4). **(I)** Gene expression of cytokines related to IgA class switching, including *Il21* and *Il10*, analyzed by qPCR (WT n = 5, *Chst4*$^{-/-}$ n = 4). Data are representative of two independent experiments, and presented as the mean ± SD; NS; not significant, *$P < 0.05$ via unpaired, two-tailed *t*-tests.

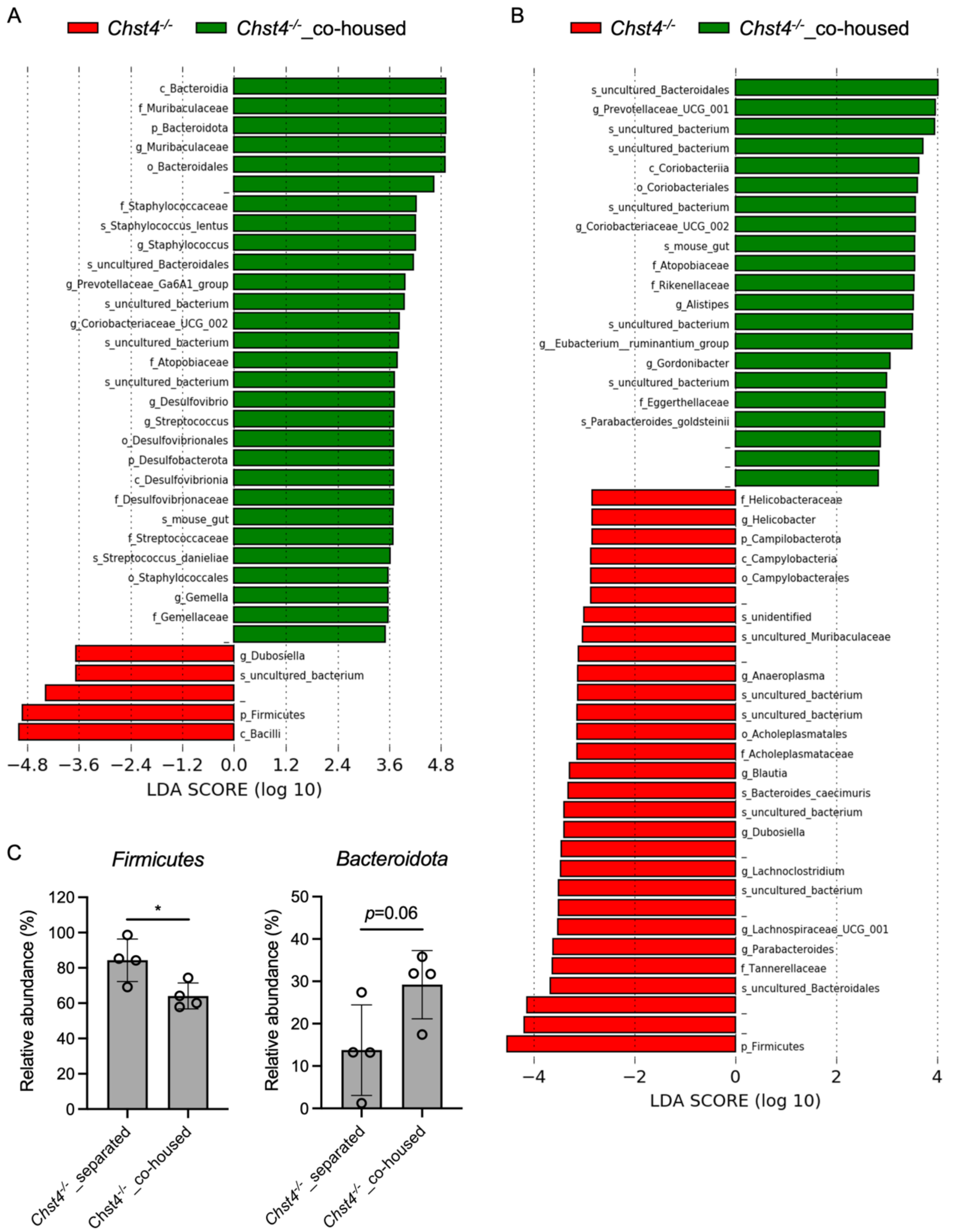

Supplemental Fig. 8. *Chst4$^{-/-}$* mice show different microbial composition after cohousing. **(A)** LEfSe analysis of ileal microbiota that were significantly different between separated and co-housed *Chst4$^{-/-}$* mice. **(B)** LEfSe analysis of fecal microbiota that were significantly different between separated and co-housed *Chst4$^{-/-}$* mice. **(C)** Relative abundance of *Firmicutes* and *Bacteroidota* in ileal samples from separated and co-housed *Chst4$^{-/-}$* mice. Data are presented as the mean ± SD; **P* < 0.05, ***P* < 0.01 via unpaired, two-tailed *t*-tests.

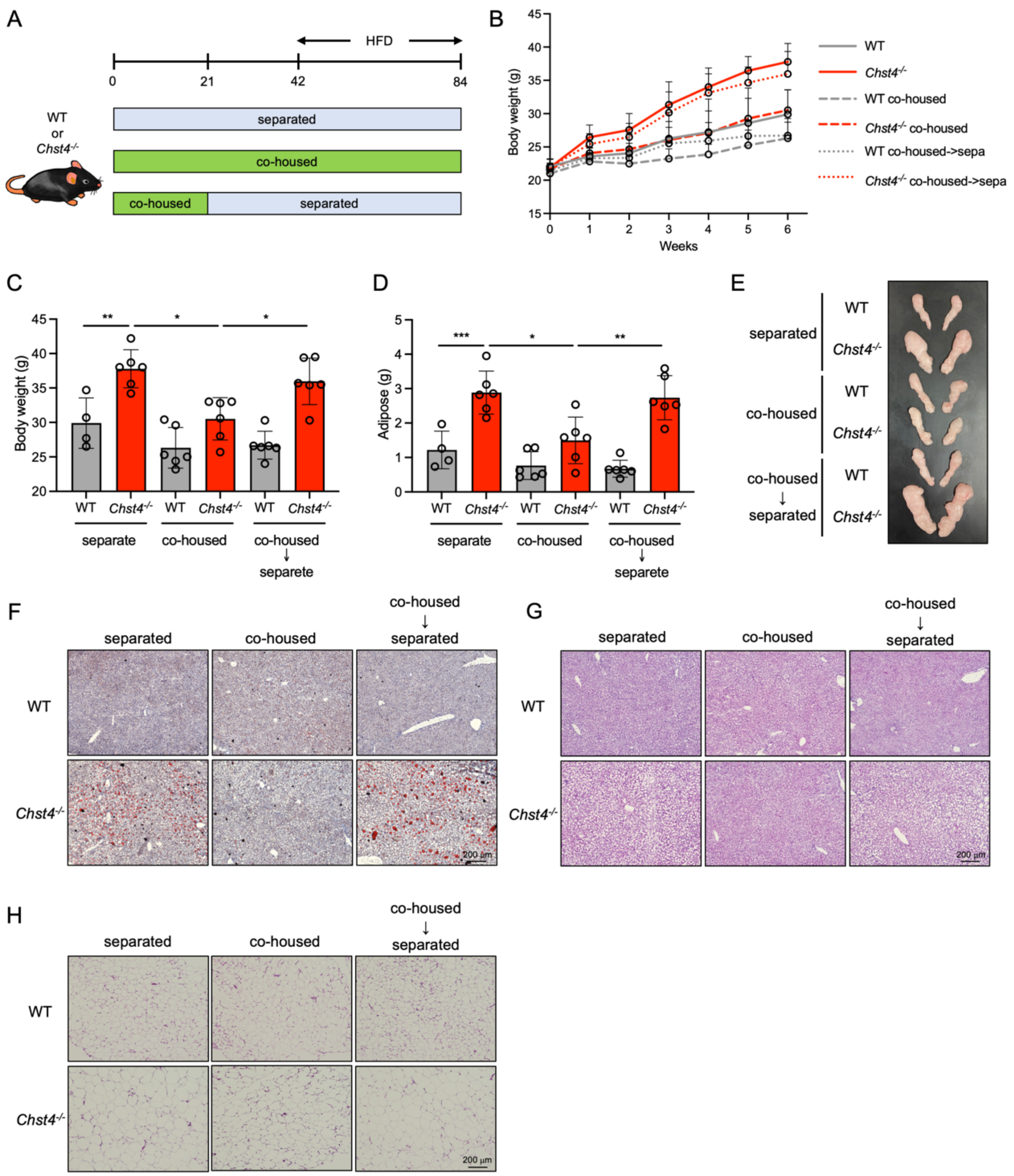

Supplemental Fig. 9. Re-isolation after co-housing increased susceptibility to HFD-induced obesity in *Chst4*$^{-/-}$ mice.

(A) The experimental schedule. **(B)** Weight gain of WT and *Chst4*$^{-/-}$ mice measured over a 6 week period. **(C)** Total weight of WT and *Chst4*$^{-/-}$ mice. **(D)** Adipose mass. **(E)** Representative image of the adipose tissues from WT and *Chst4*$^{-/-}$ mice. **(F)** Representative Oil Red O staining of the liver. **(G)** Representative HE staining of the liver. **(H)** Representative HE staining of the adipose tissues (WT n = 4, WT/co-housed n = 6, WT/co-housed→separate n = 6, *Chst4*$^{-/-}$ n = 6, *Chst4*$^{-/-}$/co-housed n = 6, *Chst4*$^{-/-}$/co-housed→separate n = 6). Data are presented as the mean ± SD; **P* < 0.05, ***P* < 0.01, ****P* < 0.001 via one-way ANOVA with multiple comparison tests.

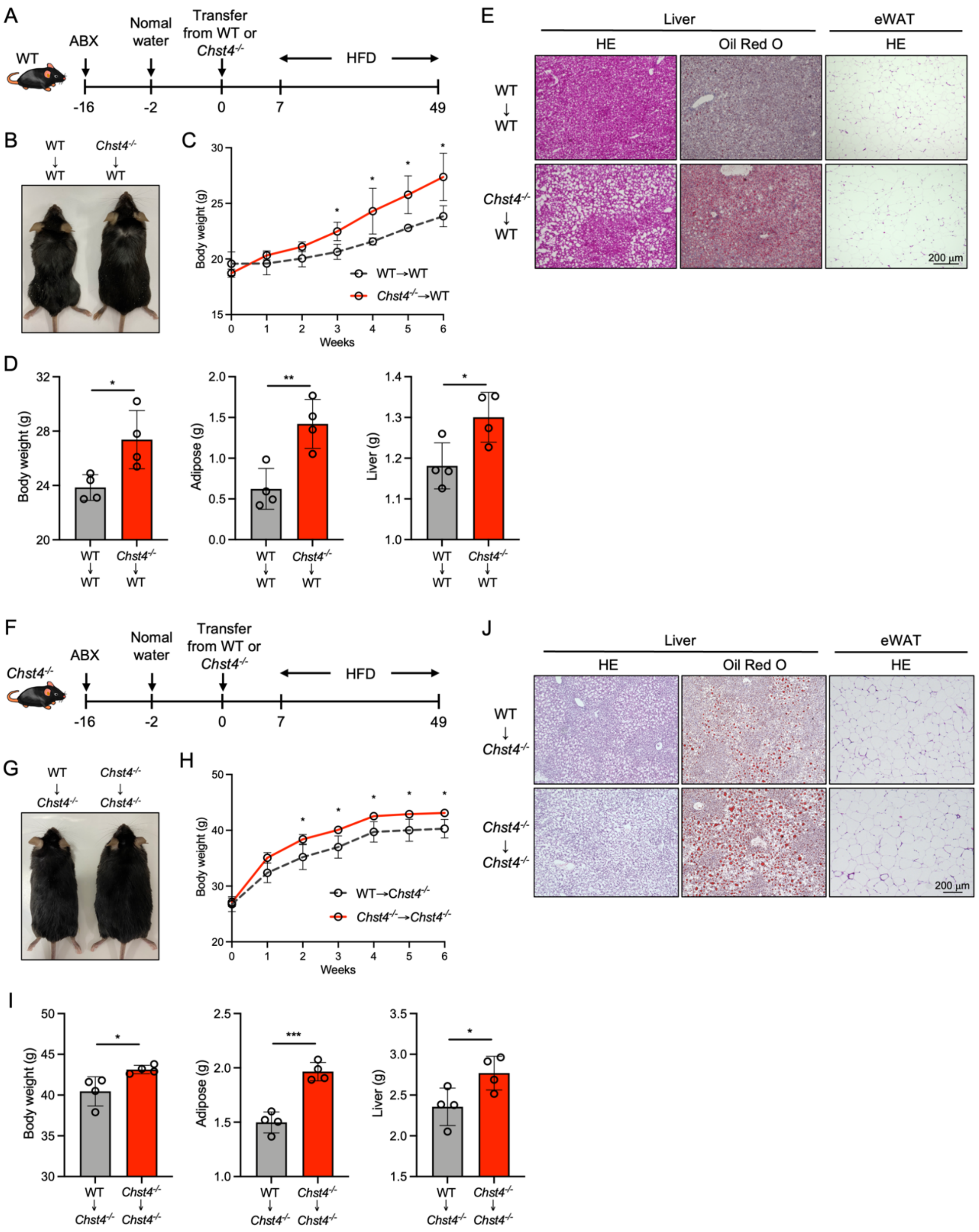

Supplemental Fig. 10. Transfer of the microbiota from *Chst4$^{-/-}$* mice induces metabolic syndrome phenotypes.

(A) Ileal contents from mice of both genotypes were isolated and suspended in PBS, followed by transfer to Abx-treated WT mice. After transfer, mice were fed an HFD, and body weight was monitored. **(B)** Weight gain measured over 6 weeks of HFD feeding. **(C)** Picture of mice transferred ileal bacteria from WT or *Chst4$^{-/-}$* mice. **(D)** Body weight, adipose, and liver weight at

6 weeks of HFD feeding. **(E)** Liver and adipose tissue were stained with HE and Oil Red O (n = 4). **(F)** **I**leal contents from mice of both genotypes were isolated and suspended in PBS, followed by transfer to Abx-treated *Chst4*$^{-/-}$ mice. After transfer, the mice were fed an HFD, and body weight was monitored. **(G)** Weight gain measured over 6 weeks of HFD feeding. **(H)** Picture of mice transferred ileal bacteria from WT or *Chst4*$^{-/-}$ mice. **(I)** Body weight, adipose, and liver weight at 6 weeks of HFD feeding. **(J)** Liver and adipose tissues were stained with HE and Oil Red O (n = 4). Data are presented as the mean ± SD; *$P < 0.05$, **$P < 0.01$ via unpaired, two-tailed *t*-tests or two-way ANOVA.
